# Next-generation sequencing of the whole mitochondrial genome identifies functionally deleterious mutations in patients with multiple sclerosis

**DOI:** 10.1371/journal.pone.0263606

**Published:** 2022-02-07

**Authors:** Ghada Al-Kafaji, Halla F. Bakheit, Faisal AlAli, Mina Fattah, Saad Alhajeri, Maram A. Alharbi, Abdulqader Daif, Manahel Mahmood Alsabbagh, Materah Salem Alwehaidah, Moiz Bakhiet

**Affiliations:** 1 Department of Molecular Medicine and Al-Jawhara Centre for Molecular Medicine, Genetics, and Inherited Disorders, College of Medicine and Medical Sciences, Arabian Gulf University, Manama, Kingdom of Bahrain; 2 Ministry of Health, Kuwait City, State of Kuwait; 3 College of Forensic Sciences, Naif Arab University for Security Sciences, Riyadh, Kingdom of Saudi Arabia; 4 King Saud University Medical City, Riyadh, Kingdom of Saudi Arabia; 5 Department of Medical Laboratory, Faculty of Allied Health, Kuwait University, Kuwait City, State of Kuwait; Tokai University School of Medicine, JAPAN

## Abstract

Multiple sclerosis (MS) is an immune-mediated disease of the central nervous system with genetics and environmental determinants. Studies focused on the neurogenetics of MS showed that mitochondrial DNA (mtDNA) mutations that can ultimately lead to mitochondrial dysfunction, alter brain energy metabolism and cause neurodegeneration. We analyzed the whole mitochondrial genome using next-generation sequencing (NGS) from 47 Saudi individuals, 23 patients with relapsing-remitting MS and 24 healthy controls to identify mtDNA disease-related mutations/variants. A large number of variants were detected in the D-loop and coding genes of mtDNA. While distinct unique variants were only present in patients or only occur in controls, a number of common variants were shared among the two groups. The prevalence of some common variants differed significantly between patients and controls, thus could be implicated in susceptibility to MS. Of the unique variants only present in the patients, 34 were missense mutations, located in different mtDNA-encoded genes. Seven of these mutations were not previously reported in MS, and predicted to be deleterious with considerable impacts on the functions and structures of encoded-proteins and may play a role in the pathogenesis of MS. These include two heteroplasmic mutations namely 10237T>C in *MT-ND3* gene and 15884G>C in *MT-CYB* gene; and three homoplasmic mutations namely 9288A>G in *MT-CO3* gene, 14484T>C in *MT-ND6* gene, 15431G>A in *MT-CYB* gene, 8490T>C in *MT-ATP8* gene and 5437C>T in *MT-ND2* gene. Notably some patients harboured multiple mutations while other patients carried the same mutations. This study is the first to sequence the entire mitochondrial genome in MS patients in an Arab population. Our results expanded the mutational spectrum of mtDNA variants in MS and highlighted the efficiency of NGS in population-specific mtDNA variant discovery. Further investigations in a larger cohort are warranted to confirm the role of mtDNA MS.

## Introduction

Multiple sclerosis (MS) is a progressive inflammatory, immune-mediated disease of the central nervous system characterized by demyelination, axonal loss and neurodegeneration [1]. MS is the most disabling neurological condition of young adults [2] and more commonly occurs in females than in males [3]. There are over two million people affected with MS worldwide [4] and a significant increase in the number of MS cases in the Arab Gulf region [5, 6]. MS can be categorized based on the phase and severity of disease progression into: clinically isolated syndrome (CIS), relapsing-remitting MS (RRMS), secondary progressive MS (SPMS), and primary progressive MS (PPMS). Approximately 85% of MS patients present with RRMS, which is marked by alternate periods of acute demyelination (relapses) and periods of neurological recovery and stability (remissions). After a period of 15–20 years, most patients with RRMS progress into a SPMS with worsened neurological function and signified by few or no acute relapses [7].

Etiologically, combined contributions of genetics and environmental factors are considered as necessary influences for MS risk and development [8]. In recent years, a considerable evidence placed mitochondrial dysfunction at the centre of the pathogenicity of MS in which mitochondrial DNA (mtDNA) defects, and mitochondrial structural and functional changes contribute to the disease progression [9, 10]. Additionally, some patients with Leber Hereditary Optic Neuropathy, a mitochondrial inherited disorder caused by mtDNA mutations present with MS symptoms including inflammatory demyelination [11, 12], suggesting a major role of mtDNA mutations in the predisposition to MS.

The mitochondria are important organelles present within almost all eukaryotic cells. The primary function of mitochondria is generation of energy in the form of adenosine triphosphate (ATP) through the process of oxidative phosphorylation (OXPHOS). Reactive oxygen species (ROS) are generated by mitochondria as by-products of respiration and OXPHOS. The mitochondria are unique in that they have their own genome (mtDNA), which is solely maternally inherited [13]. Human mtDNA is a double-stranded circular molecule of about 16,500 nucleotides and contains 37 genes coding for 22 tRNAs and 2 rRNAs (12S, 16S) which are essential for protein synthesis within mitochondria, and 13 protein subunits of the electron transport chain (ETC) complexes, all of which are involved in the OXPHOS. The 13 mtDNA gene-encoded proteins include seven subunits of NADH dehydrogenase of complex I (ND1, ND2, ND3, ND4, ND4L, ND5 and ND6); cytochrome b-c1 (CYB) of ubiquinol-cytochrome c reductase of complex III; three subunits of cytochrome c oxidase of complex IV (COX I, COX II and COX III); and two subunits of mitochondrial ATP synthase of complex V (ATP6 and ATP8). In addition, the mitochondrial control region (or D-loop) is a non-coding region and contains essential transcription and replication elements responsible for the expression of mitochondrial genome. In human, the mtDNA is present in multiple copies per cell, which gives rise to an important feature of mitochondrial genetics; homoplasmy (when all copies of mtDNA are identical) and heteroplasmy (when there is a mixture of normal and mutated mtDNA copies) [14].

It is well documented that the mtDNA is more susceptible to oxidative damage and acquires higher rates of mutation than the nuclear DNA (nDNA), possibly due to absence of protective histone, inadequate DNA repair pathways and high ROS exposure [15]. Mutations in the mtDNA are known to cause diverse mitochondrial disorders, and are also linked to other complex traits such as ageing, cancer and neurodegenerative diseases, all are associated with defects in oxidative energy metabolism [16, 17]. Specifically, impaired mitochondrial energy metabolism is considered as a key pathogenic factor in MS and plays an important role in axonal degeneration [9, 10, 18]. From this perspective, neurogenetic studies in relation to mtDNA provided an evidence of the implication of mtDNA mutations/variants in the pathogenesis or risk of MS. Specifically, variants in mtDNA-encoded complex I have been linked to MS in different ethnic populations [19, 20]. In our previous study in Saudi patients with MS, we identified four novel mutations in the mtDNA-encoded *ND4* gene, which reduced stability of complex I [21]. However, most of the studies on MS have used targeted sequencing for specific mtDNA genes/ gene regions of interest [19–21] or selected mitochondrial polymorphisms for disease association [22–24]. While analysis of whole mtDNA genome highlighted specific haplogroup-defining variants in Caucasian patients with MS, but did not examine variants across the mitochondrial genome [25].

In this study, we aimed to analyze the whole mitochondrial genome using next-generation sequencing (NGS) from blood samples of Saudi subjects with RRMS and healthy controls to identify mtDNA disease-related mutations/variants. We hypothesized that mtDNA mutations/variants would occur at higher rate in cases compared to controls and implicated in susceptibility to MS. We also hypothesized that mutations in mtDNA-encoded genes occur in cases to a deleterious level and play a role in the pathogenesis of MS. NGS data can yield a large degree of population-specific mtDNA variants and permit extensive interpretation of potential disease-causing mutations.

## Methods

### Subjects

A cohort of 47 Saudi individuals consisting 23 patients with RRMS and 24 healthy controls were included in this study. Written consent forms were obtained from each participant. The contents of the consent forms and the study were reviewed and approved by the Scientific and Ethics Committee in King Saud University, College of Medicine, Kingdom of Saudi Arabia (KSA), and the Medical Research and Ethics Committee in the College of Medicine and Medical Sciences, Arabian Gulf University, Kingdom of Bahrain. The RRMS patients were recruited from the Neurology Outpatient Clinic at King Khalid Hospital, King Saud University, KSA. All patients were assessed independently by a neurologist and the diagnosis of RRMS was done according to the McDonald criteria [26] with at least two previous relapses in CNS regions, confirmed by a neurological examination, a magnetic resonance imaging (MRI), and electrophysiological studies. The healthy control individuals who had no neurological conditions or history of autoimmune and inflammatory disease were enrolled in this study from King Khalid hospital Blood Bank, KSA. Demographic and clinical data including age, gender, body mass index (BMI) and blood pressure were recorded for both patients and controls. Disease duration, disability status, and medications were recorded for all patients. The disability status was evaluated using the Kurtzke Expanded Disability Status Scale (EDSS).

### Extraction of genomic DNA

Peripheral blood samples (5 ml) from patients and healthy controls were collected in ethylenediaminetetraacetic acid (EDTA) tubes. Genomic DNA was extracted from the peripheral blood using the QIAMP DSP DNA kit (Qiagen, Hilden, Germany) as previously described [21]. Briefly, 200 μl blood was mixed with 20 μl protease and 200 μl of lysis buffer. The mixture was incubated at 56°C for 10 min, and centrifuged at 20,000 x g for 1 min at 4°C. Then 200 μl of absolute ethanol was added and centrifuged at 6000 ×*g* for 1 min. This was followed by washing steps using 500 μl of washing buffer and centrifugation at 6,000 x g for 1 min and then at 20,000 x g for 3 min at room temperature. For genomic DNA elution, 200 μl of elution buffer was added, incubated at room temperature for 1 min and centrifuged at 6,000 x g for 1 min at room temperature. For quality control, all DNA samples were quantified and checked for purity using the NanoDrop ND-1000 ultraviolet-visible light spectrophotometer (Thermo Fisher Scientic, Inc.).

### Amplicon generation

The mtDNA of all genomic DNA samples were amplified using long-range PCR (Qiagen Long range PCR kit, Cat# 206403) with two sets of primers: Primer Set 1, Forward L644–GACGGGCTCACATCACCCCATAA, and Reverse H8982-GCGTACGGCCAGGGCTATTGGT. Primer Set 2, Forward L8789-GCCACAACTAACCTCCTCGGACTCCT and Reverse H877- GGTGGCTGGCACGAAATTGACC. A 50 ng DNA was taken as input for the PCR amplifications. The amplified products were checked using 1% Agarose gel electrophoresis.

### Next generation sequencing of whole mitochondrial genome

Library preparation and purity were performed according to the manufacturer’s protocol. Briefly, PCR products amplified from both primer sets were pooled together. The KAPA HTP Library Preparation Kit (Roche NimbleGen) was used to prepare libraries for whole genome sequencing. First, the DNA was fragmented to ~250 bp using Covaris M220 Focused-Ultrasonicator and the sheared DNA was checked on TapeStation using the HSD1000 DNA ScreenTapes (Agilent) for fragment distribution. Sheared DNA was then end repaired and adapter ligated in a series of enzymatic steps. The Adapter ligated products were then purified by Agencourt AmpureXP Beads (BeckmanCoulter) followed by size selection and then enriched using the following thermal conditions: Initial denaturation 98°C for 45 sec; 6 cycles of 98°C for 15 sec, 60°C for 30 sec,72°C 30 sec; final extension of 72°C for 1 min. PCR products were then purified by Agencourt AmpureXP Beads (BeckmanCoulter) and the final libraries were checked for fragment size distribution on TapeStation using the D1000 DNA ScreenTapes (Agilent). Prepared libraries were quantified using the Qubit HS Assay (Invitrogen). The obtained libraries were pooled and diluted to final optimal loading concentration before cluster amplification on Illumina flow cell. Once the cluster generation was completed, the cluster flow cell was loaded on Illumina HiSeq X instrument following the manufacturer’s instructions (Illumina) to generate 150 bp paired end reads.

### Sequence data analysis

Paired end sequencing data were exported to FASTQ file. Sequence reads were trimmed using custom script to remove adapters and bases where the quality value was less than 20. The trimmed reads were aligned using Burrows–Wheeler Aligner (BWA) aligner. Alignment was performed to hg19 version of the genome available from UCSC genome browser. The aligned reads were filtered for low-quality mapping score, unusual insert-size and cross-chromosome mapping. Reads were re-aligned around variants and Base Quality Score Recalibration (BQSR) was done using GATK-lite program. Revised Cambridge Reference Sequence (rCRS) of the Human Mitochondrial DNA (NC_012920.1) was applied as reference mitochondrial sequence. Variants were annotated using MITOMAP and Human Mitochondrial Genome Database (mtDB). A total sequencing coverage of 10,000 X of the mitochondrial genome for this work was achieved. At such coverage heteroplasmy at 5% levels would be detectable. We determined whether the mutations were novel or known by investigating their presence in the MITOMAP, mtDB, ClinVar databases as well as Google search for individual studies reporting mtDNA mutations.

### Functional and structural prediction analysis

The effect of mutations-caused amino acid changes on protein function was predicted by a combination of tools that use sequence homology, evolutionary conservation, and protein structural information [27]. These tools include:

1. Polymorphism Phenotyping (PolyPhen): Prediction based on protein sequence alignment and structure-based method, and uses a machine learning classification to categorize variants as benign, possibly damaging, and probably damaging with scores of < 0.446, > 0.446, and > 0.908 respectively.
2. Sorting Intolerant From Tolerant (SIFT): Uses sequence homology based on multiple sequence alignment conservation approach and how an amino acid substitution affects protein function. SIFT categories are benign and tolerated with scores of > 0.05 and < 0.05 respectively.
3. Combined Annotation Dependent Depletion (CADD): Integrates multiple information sources including conservation, structure-based features and functional information, and uses a machine learning approach to categorize variants as benign or deleterious. CADD scores range from 1–99 (variants with higher scores are more likely to be deleterious).
4. Mutation Assessor: Prediction based on evolutionary conservation of the affected amino acid in protein homologs. Mutation Assessor categories are neutral, low, medium and high with score ranging from 0–1 (variants with higher scores are more likely to be deleterious).

Moreover, the impact of single nucleotide substitutions-caused amino acid changes on three-dimensional (3-D) protein structure was determined using PyMOL (Molecular Graphics System Version 2.0, Schrodinger LLC). The 3-D protein structure was retrieved from https://www.ebi.ac.uk/pdbe.

### Statistical analysis

The SPSS software (version 23; IBM Corp., Armonk, NY, USA) was used for data analysis. For differences in the demographic and clinical parameters between the patients and healthy controls, the Kolmogorov-Smirnov test was first used to evaluate the normal distribution of the data. Then the comparisons of variables between the two groups were done using a Chi-square test for categorical variables or equivalent non-parametric Wilcoxon signed-rank test and Mann-Whitney test for normally distributed variables. Fischer exact test was used to assess the differences in variant frequencies between patients and controls. Chi-Square test was used to determine the association of variants in patients and controls and the odds ratios (OD), relative risk (RR) and 95% confidence interval (95% CI) were reported. A two sided P value < 0.05 was considered to be statistically significant.

## Results

### Demographic and clinical data

Table 1 shows the demographic and clinical data of RRMS patients (n = 23) and healthy control individuals (n = 24). The mean age of patients was 28 years (standard deviation [SD], 7.5) and ranged from 18–44 years. The mean age of healthy controls was 31 years (SD, 7.5) and ranged from 22–52 years. The gender ratio (Male:Female; M:F) of patient was 5M:18F and the gender ratio of healthy controls was 5M:19F. There was no significant difference in the mean age (P = 0.14) or sex distribution between the two groups (P = 0.66). Also no significant differences were found between the two groups for mean BMI (P = 0.23) or mean systolic blood pressure (P = 0.16) and mean diastolic blood pressure (P = 0.37). The mean disease duration for the patient group was 5.3 years (SD, 4) and ranged from 1–15 years. The mean EDSS for the patient group was 3.9 (SD, 1.4) and ranged from 2–6.5. The patients were under the following treatment: of Avonex, Betaferon, Glienya, Rebif and Tysabri (n = 3, 8, 3, 5 and 4 respectively).

**Table 1:** Demographic and clinical data of RRMS patients and healthy controls.

	Patients (n = 23)	Control (n = 24)
Age		
Mean ± SD	28 ± 7.5	31 ± 7.5
Range years	18–44	22–52
Sex		
Male	5	5
Female	18	19
BMI		
Mean ± SD	27 ± 6.2	29 ± 5.5
Blood pressure		
Systolic		
Mean ± SD	123 ± 11.4	131 ± 16.4
Diastolic		
Mean ± SD	72 ± 11.9	76 ± 9.6
Disease duration		
Mean ± SD	5.3 ± 4.0	
Range	1–15	
EDSS		
Mean ± SD	3.9 ± 1.4	
Range	2–6.5	
Medication		
Avonex	3	
Betaferon	8	
Glienya	3	
Rebif	5	
Tysabri	4	

Data are presented as number or mean ± standard deviation (SD). RRMS, relapse-remitting multiple sclerosis; EDSS, Expanded Disability Status Scale.

### Next generation sequencing of whole mitochondrial genome

We analyzed the whole mitochondrial genome from 47 subjects, including 23 RRMS patients and 24 healthy controls using NGS. All 47 mtDNA sequences passed quality controls. The overall alignment and the passed alignment percentage (alignment to hg19) in all samples were around 94.29% and 85.20% respectively. The average coverage of the samples on the panel was around 99.54% and the on-target percentage for the samples was around 86.72%. The data sets of mtDNA sequences generated in the present study were deposited at NCBI (http://www.ncbi.nlm.nih.gov/), Sequence Read Archive Repository (accession SRA data ref. no. PRJNA781092). The reference BioSample accession numbers are SAMN23235107 and SAMN23235153.

### Distribution of variants in mitochondrial genome among patients and controls

**Fig 1:**
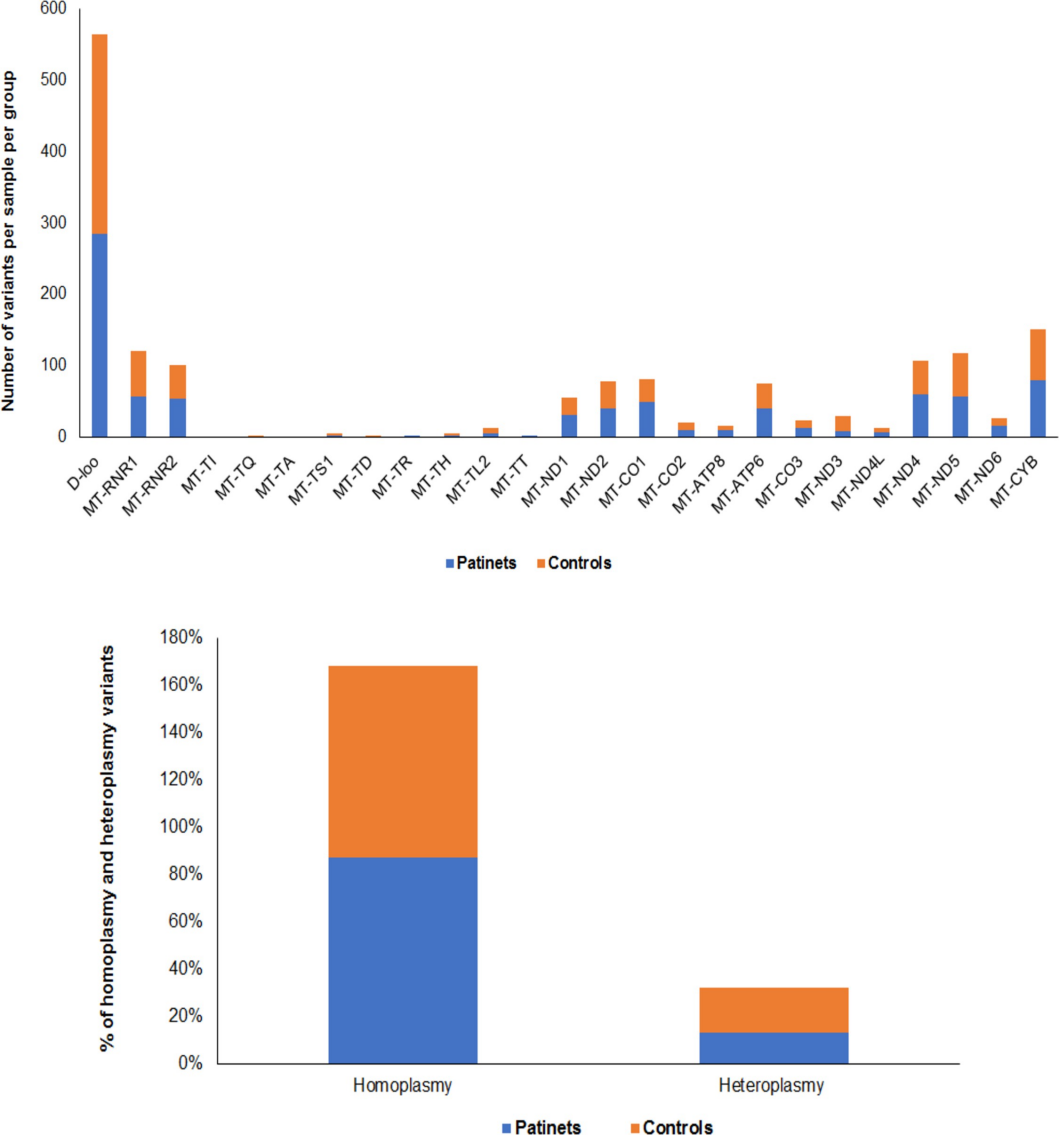
**A**. Distribution of variants across mitochondrial genome in RRMS patients and healthy controls. A large number of variants were located in the D-loop region, and the total number of coding and non-coding variants were higher in patients than in controls. **B**. Percentage (%) of homoplasmic and heteroplasmic variants in RRMS patients and healthy controls. The majority of mtDNA variants across all samples were homoplasmic (85% in patients and 86% in controls) as compared with the heteroplasmic variants (15% in patients and 14% in controls).

Overall 3248 mtDNA variants were found across all samples. On average, 69 variants were detected per sample. Total number of variants detected in patients was higher as compared to controls. Of total, 1686 variants were found in patients and 1562 variants were found in controls. The variants were compared at the genomic position level for patients and controls, and were found to be distributed around all regions of mitochondrial genome in the two groups (Fig 1A). The difference in variant distribution across mitochondrial genome indicates that a large number of variants were located in the D-loop region, and the total number of coding and non-coding variants were higher in patients than in controls (Fig 1A). The majority of mtDNA variants across all samples were homoplasmic (85% in patients and 86% in controls) as compared with the heteroplasmic variants (15% in patients and 14% in controls) (Fig 1B).

### Unique and shared mtDNA variants in patients and controls

**Fig 2:**
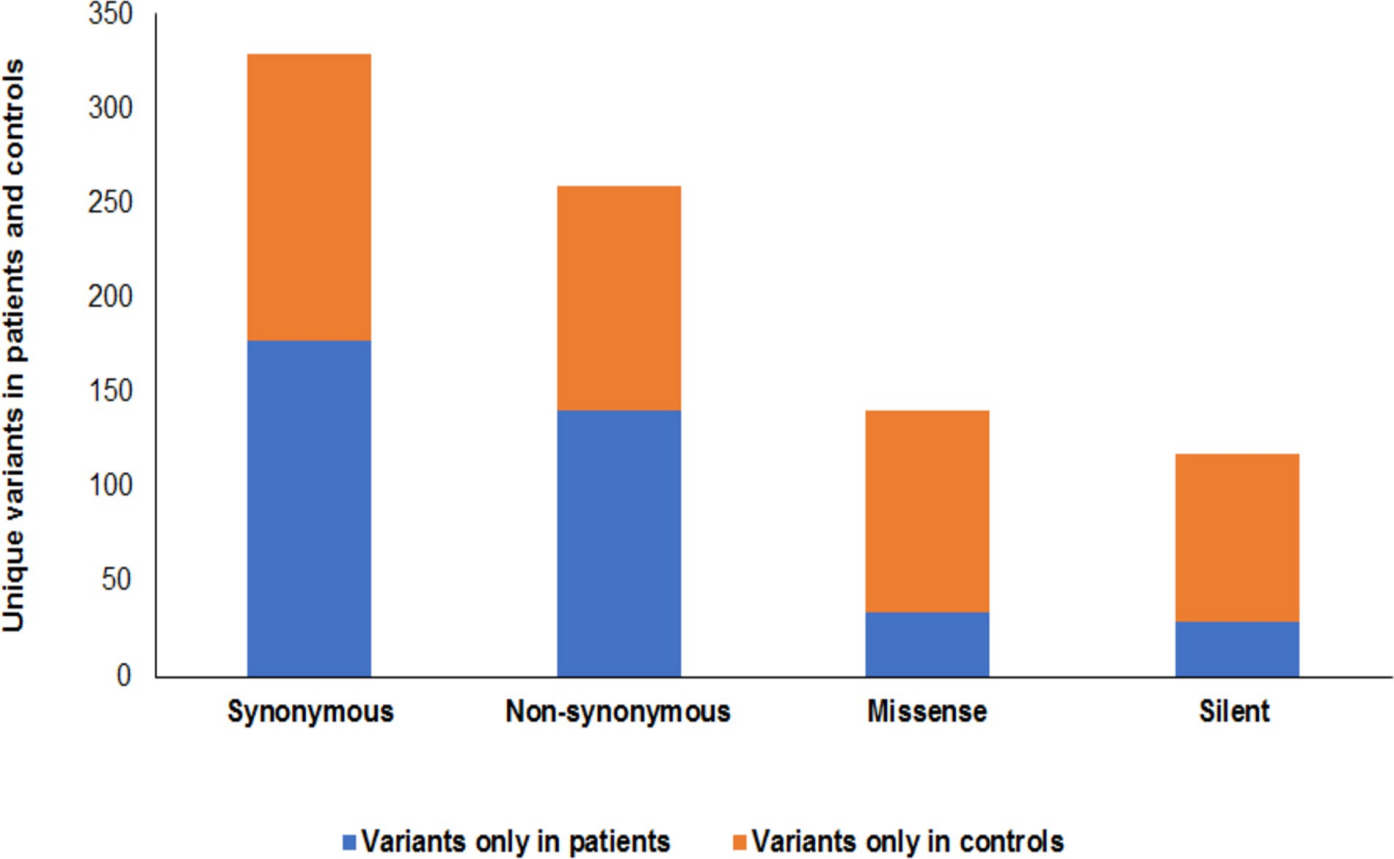
Unique variants in RRMS patients and healthy controls. The unique variant only found in patients include 177 synonymous and 141 nonsynonymous (34 missense and 107 silent). The unique variant only found in controls include 152 synonymous and 118 nonsynonymous (29 missense and 89 silent) variants.

Comparison of mtDNA variants among patients and controls revealed a number of unique variants only observed in patients or only observed in controls, which occurred in one or multiple samples. Of the unique variants in patients, 177 were synonymous and 141 were nonsynonymous including 34 missense and 107 silent variants (Fig 2). Of the unique variants observed in controls, 152 were synonymous and 118 were nonsynonymous including 29 missense and 89 silent variants (Fig 2). Details of these variants are displayed in Table 2.

**Table 2:** mtDNA variants observed only in RRMS patients or only in healthy controls.

	Patients					Controls				
Locus	Nucleotide change	Amino acid change	Type of variant	Nature	Variant ID	Nucleotide change	Amino acid change	Type of variant	Nature	Variant ID
D-loop	58T>C	-	Substitution	Heteroplasmy	NP	56insT	-	Insertion	Homoplasmy	NP
D-loop	153A>G	-	Substitution	Homoplasmy	NP	143G>A	-	Substitution	Homoplasmy	NP
D-loop	185G>A	-	Substitution	Homoplasmy	NP	182C>T	-	Substitution	Homoplasmy	NP
D-loop	189A>G	-	Substitution	Heteroplasmy	NP	183A>G	-	Substitution	Homoplasmy	NP
D-loop	196T>C	-	Substitution	Homoplasmy	NP	188insGC	-	Insertion	Heteroplasmy	NP
D-loop	200A>G	-	Substitution	Homoplasmy	NP	193A>C	-	Substitution	Heteroplasmy	NP
D-loop	217T>C	-	Substitution	Homoplasmy	NP	194delTT	-	Deletion	Heteroplasmy	NP
D-loop	236T>C	-	Substitution	Homoplasmy	NP	199T>C	-	Substitution	Homoplasmy	NP
D-loop	242C>T	-	Substitution	Homoplasmy	NP	228G>A	-	Substitution	Homoplasmy	NP
D-loop	247G>A	-	Substitution	Homoplasmy	NP	239T>C	-	Substitution	Homoplasmy	NP
D-loop	282T>C	-	Substitution	Homoplasmy	NP	324C>T	-	Substitution	Homoplasmy	NP
D-loop	309delC	-	Deletion	Heteroplasmy	NP	480T>C	-	Substitution	Homoplasmy	NP
D-loop	340C>T	-	Substitution	Homoplasmy	NP	482T>C	-	Substitution	Heteroplasmy	NP
D-loop	497C>T	-	Substitution	Homoplasmy	NP	511C>T	-	Substitution	homoplasmy	NP
D-loop	499G>A	-	Substitution	Homoplasmy	NP	567insCC	-	Insertion	Homoplasmy	NP
D-loop	16114C>A	-	Substitution	Homoplasmy	NP	16067C>T	-	Substitution	Homoplasmy	NP
D-loop	16148C>T	-	Substitution	Homoplasmy	NP	16136T>C	-	Substitution	Homoplasmy	NP
D-loop	16158A>G	-	Substitution	Homoplasmy	NP	16147C>G	-	Substitution	Homoplasmy	NP
D-loop	16163A>G	-	Substitution	Homoplasmy	NP	16169C>T	-	Substitution	Homoplasmy	NP
D-loop	16168C>T	-	Substitution	Homoplasmy	NP	16184C>T	-	Substitution	Homoplasmy	NP
D-loop	16185C>T	-	Substitution	Homoplasmy	NP	16219A>G	-	Substitution	Homoplasmy	NP
D-loop	16186C>T	-	Substitution	Homoplasmy	NP	16232C>A	-	Substitution	Homoplasmy	NP
D-loop	16193C>T	-	Substitution	Homoplasmy	NP	16235A>G	-	Substitution	Heteroplasmy	NP
D-loop	16218C>T	-	Substitution	Homoplasmy	NP	16239C>T	-	Substitution	Homoplasmy	NP
D-loop	16230A>G	-	Substitution	Homoplasmy	NP	16248C>T	-	Substitution	Homoplasmy	NP
D-loop	16234C>T	-	Substitution	Homoplasmy	NP	16249T>C	-	Substitution	Homoplasmy	NP
D-loop	16257C>T	-	Substitution	Homoplasmy	NP	16256C>T	-	Substitution	Homoplasmy	NP
D-loop	16259C>T	-	Substitution	Homoplasmy	NP	16270C>T	-	Substitution	Homoplasmy	NP
D-loop	16260C>T	-	Substitution	Homoplasmy	NP	16286C>T	-	Substitution	Hetero	NP
D-loop	16263T>C	-	Substitution	Homoplasmy	NP	16309A>G	-	Substitution	Homoplasmy	NP
D-loop	16269A>G	-	Substitution	Homoplasmy	NP	16327C>A	-	Substitution	Homoplasmy	NP
D-loop	16284A>G	-	Substitution	Heteroplasmy	NP	16390G>A	-	Substitution	Homoplasmy	NP
D-loop	16290C>T	-	Substitution	Homoplasmy	NP	16482A>G	-	Substitution	Homoplasmy	NP
D-loop	16293A>G	-	Substitution	Homoplasmy	NP					
D-loop	16356T>C	-	Substitution	Heteroplasmy	NP					
rRNA genes										
*MT-RNR1* s-rRNA	825T>A	-	Substitution	Homoplasmy	NP	669T>C	-	Substitution	Homoplasmy	NP
*MT-RNR1* s-rRNA	827A>G	-	Substitution	Heteroplasmy	rs28358569	769G>A	-	Substitution	Homoplasmy	NP
*MT-RNR1* s-rRNA	955iinsCC	-	Insertion	Heteroplasmy	NP					
*MT-RNR1* s-rRNA	961T>C	-	Substitution	Heteroplasmy	NP	1243T>C	-	Substitution	Homoplasmy	rs28358572
*MT-RNR1* s-rRNA	1007G>A	-	Substitution	Homoplasmy	rs111033213					
*MT-RNR1* s-rRNA	1048C>T	-	Substitution	Homoplasmy	NP	1406T>C	-	Substitution	Homoplasmy	rs111033322
*MT-RNR2* l-rRNA	1762A>G	-	Substitution	Homoplasmy	NP					
*MT-RNR2* l-rRNA	1812C>T	-	Substitution	Homoplasmy	NP	2416T>C	-	Substitution	Homoplasmy	NP
*MT-RNR2* l-rRNA	1888G>A	-	Substitution	Homoplasmy	NP	2626T>C	-	Substitution	Homoplasmy	NP
*MT-RNR2* l-rRNA	1927G>A	-	Substitution	Heteroplasmy	NP	2702G>A	-	Substitution	Homoplasmy	NP
*MT-RNR2* l-rRNA	2245A>G	-	Substitution	Homoplasmy	NP					
*MT-RNR2* l-rRNA	2283C>T	-	Substitution	Heteroplasmy	NP	2789C>T	-	Substitution	Homoplasmy	NP
*MT-RNR2* l-rRNA						3197T>C	-	Substitution	Homoplasmy	NP
*MT-RNR2* l-rRNA	2648T>C	-	Substitution	Heteroplasmy	NP					
*MT-RNR2* l-rRNA	2885T>C	-	Substitution	Heteroplasmy	NP					
*MT-RNR2* l-rRNA	3184C>T	-	Substitution	Homoplasmy	NP					
tRNA genes										
*MT-TI* tRNA-Leu	4312C>T	-	Substitution	Homoplasmy	NP					
*MT-TA* tRNA-Ala	5603C>T	-	Substitution	Homoplasmy	NP					
*MT-TS1* tRNA-Ser	7471delA	-	Deletion	Homoplasmy	NP					
*MT-TR* tRNA-Arg	10410T>C	-	Substitution	Homoplasmy	NP					
*MT-TR* tRNA-Arg	10463T>C	-	Substitution	Homoplasmy	NP					
*MT-TH* tRNA-His						12172A>G	-	Substitution	Homoplasmy	NP
*MT-TH* tRNA-His						12192G>A	-	Substitution	Homoplasmy	rs3134560
*MT-TL2* tRNA-Leu						12295T>C	-	Substitution	Homoplasmy	NP
*MT-TT* tRNA-Thr	15907A>G	-	Substitution	Homoplasmy	NP					
*MT-TT* tRNA-Thr	15928G>A	-	Substitution	Homoplasmy	NP					
Protein-coding genes										
*MT-ND1*	3316G>A	p.Ala4Thr	Missense	Homoplasmy	NP	3348A>G	p.Leu14 (=)	Silent	Heteroplasmy	NP
*MT-ND1*	3438G>A	p.Gly44 (=)	Silent	Homoplasmy	NP	3394T>C	p.Tyr30His	Missense	Homoplasmy	rs41460449
*MT-ND1*				Homoplasmy		3411A>G	p.Lys35 (=)	Silent	Homoplasmy	NP
*MT-ND1*	3516C>A	p.Leu70 (=)	Silent	Homoplasmy	NP	3421G>A	p.Val39Ile	Silent	Homoplasmy	NP
*MT-ND1*	3533C>T	p.Thr76Ile	Missense	Homoplasmy	rs377091327	3505A>G	p.Thr67Ala	Missense	Homoplasmy	NP
*MT-ND1*	3720A>G	p.Gln138 (=)	Silent	Homoplasmy	NP	3654C>T	p.Ile116 (=)	Silent	Homoplasmy	NP
*MT-ND1*	3768A>G	p.Leu154 (=)	Silent	Heteroplasmy	NP	3915G>A	p.Gly203 (=)	Silent	Homoplasmy	NP
*MT-ND1*	3834G>A	p.Leu176 (=)	Silent	Heteroplasmy	NP					
*MT-ND1*	3865A>G	p.Ile187Val	Missense	Heteroplasmy	NP					
*MT-ND1*	3944T>C	p.Ile213Thr	Missense	Heteroplasmy	NP					
*MT-ND1*	3948A>G	p.Glu214 (=)	Silent	Homoplasmy	NP					
*MT-ND2*	4586T>C	p.Ala39 (=)	Silent	Homoplasmy	NP	4509T>C	p.Phe14Leu	Missense	Homoplasmy	NP
*MT-ND2*	4646T>C	p.Tyr59 (=)	Silent	Heteroplasmy	NP	4727A>G	p.Met86 (=)	Silent	Homoplasmy	NP
*MT-ND2*	4695T>C	p.Phe76Leu	Missense	Homoplasmy	NP	4796A>G	p.Ala109 (=)	Silent	Homoplasmy	NP
*MT-ND2*	4917A>G	p.Asn150Asp	Missense	Homoplasmy	NP	4823T>C	p.Val118 (=)	Silent	Homoplasmy	NP
*MT-ND2*	5073A>G	p.Ile202Val	Missense	Homoplasmy	NP	4824A>G	p.Thr119Ala	Missense	Homoplasmy	rs15564229
*MT-ND2*	5096T>C	p.Ile209 (=)	Silent	Homoplasmy	NP	4973T>C	p.Gly168 (=)	Silent	Heteroplasmy	NP
*MT-ND2*	5120A>G	p.Leu217 (=)	Silent	Homoplasmy	NP	5246C>A	p.Gly259 (=)	Silent	Homoplasmy	NP
*MT-ND2*	5191C>T	p.Thr241Met	Missense	Homoplasmy	NP	5263C>T	p.Ala265Val	Missense	Homoplasmy	NP
*MT-ND2*	5231G>A	p.Leu254 (=)	Silent	Homoplasmy	NP	5294C>T	p.Ser275 (=)	Silent	Homoplasmy	NP
*MT-ND2*	5333T>C	p.Leu288 (=)	Silent	Homoplasmy	NP	5315A>G	p.Met282 (=)	Silent	Homoplasmy	NP
*MT-ND2*	5390A>G	p.Met307 (=)	Silent	Homoplasmy	NP	5480A>G	p.Leu337 (=)	Silent	Heteroplasmy	NP
*MT-ND2*	5426T>C	p.His319 (=)	Silent	Homoplasmy	NP					
*MT-ND2*	5437C>T	p.Thr323Ile	Missense	Homoplasmy	NP					
*MT-ND2*	5442T>C	p.Phe325Leu	Missense	Homoplasmy	NP					
*MT-CO1*	5999T>C	p.Ala32 (=)	Silent	Heteroplasmy	NP	5913G>A	p.Asp4Asn	Missense	Heteroplasmy	rs20161727
*MT-CO1*	6045C>T	p.Leu48 (=)	Silent	Homoplasmy	NP	6040A>C	p.Asn46Thr	Missense	Homoplasmy	rs1556423072
*MT-CO1*	6047A>G	p.Leu48 (=)	Silent	Heteroplasmy	NP	6080A>G	p.Thr59 (=)	Silent	Homoplasmy	NP
*MT-CO1*	6152T>C	p.Val83 (=)	Silent	Homoplasmy	NP	6248T>C	p.Ser115 (=)	Silent	Homoplasmy	NP
*MT-CO1*	6185T>C	p.Phe94 (=)	Silent	Homoplasmy	NP	6497T>C	p.Ser198 (=)	Silent	Homoplasmy	NP
*MT-CO1*	6386C>T	p.Ala161 (=)	Silent	Heteroplasmy	NP	6663A>G	p.Ile254Val	Missense	Homoplasmy	rs200784106
*MT-CO1*	6524T>C	p.Ala161 (=)	Silent	Heteroplasmy	NP	6707T>C	p.Phe268 (=)	Silent	Homoplasmy	NP
*MT-CO1*	6546C>T	p.Leu215Phe	Missense	Homoplasmy	rs1603220531	6722G>A	p.Met273 (=)	Silent	Homoplasmy	NP
*MT-CO1*	6599A>G	p.Gln232 (=)	Silent	Homoplasmy	NP	7175T>C	p.Thr424 (=)	Silent	Homoplasmy	rs28358874
*MT-CO1*	6746C>T	p.Gly281 (=)	Silent	Homoplasmy	NP	7274C>T	p.Gly457 (=)	Silent	Homoplasmy	NP
*MT-CO1*	7146A>G	p.Thr415Ala	Missense	Homoplasmy	rs372136420	7394A>G	p.Gly497 (=)	Silent	Homoplasmy	NP
*MT-CO2*	7645T>C	p.Leu20 (=)	Silent	Heteroplasmy	NP	7771A>G	p.Glu62 (=)	Silent	Homoplasmy	NP
*MT-CO2*	7681C>T	p.Phe32 (=)	Silent	homoplasmy	NP	7853G>A	p.Val90Ile	Missense	Homoplasmy	NP
*MT-CO2*	7762G>A	p.Gln59 (=)	Silent	Homoplasmy	NP	8014A>T	p.Val143 (=)	Silent	Homoplasmy	NP
*MT-CO2*	7789G>A	p.Leu68 (=)	Silent	Homoplasmy	rs386829014	8206G>A	p.Met207 (=)	Silent	Homoplasmy	NP
*MT-CO2*	7963A>G	p.Leu126 (=)	Silent	Homoplasmy	NP	8251G>A	p.Gly222 (=)	Silent	Homoplasmy	NP
*MT-CO2*	8143T>C	p.Ala186 (=)	Silent	Homoplasmy	NP	8374A>G	p.Gln3 (=)	Silent	Homoplasmy	NP
*MT-CO2*	8155G>A	p.Gly190 (=)	Silent	Heteroplasmy	NP					
*MT-CO2*	8222T>C	p.Leu213 (=)	Silent	Homoplasmy	NP					
*MT-ATP8*	8428C>T	p.Phe21 (=)	Silent	Homoplasmy	NP	8448T>C	p.Met28Thr	Missense	Homoplasmy	rs879056797
*MT-ATP8*	8468C>T	p.Leu35 (=)	Silent	Homoplasmy	NP	8521A>G	p.Glu52 (=)	Silent	Homoplasmy	NP
*MT-ATP8*	8490T>C	p.Met42Thr	Missense	Homoplasmy	rs1603221530	8723G>A	p.Arg66Gln	Missense	Homoplasmy	
*MT-ATP6*	8566A>G	p.Ile14Val	Missense	Homoplasmy	NP	8901A>G	p.Leu125 (=)	Silent	Homoplasmy	NP
*MT-ATP6*	8655C>T	p.Ile43 (=)	Silent	Homoplasmy	NP	8994G>A	p.Leu156 (=)	Silent	Homoplasmy	NP
*MT-ATP6*	8658C>T	p.Thr44 (=)	Silent	Hetero	NP	8793T>C	p.Pro89 (=)	Silent	Heteroplasmy	NP
*MT-ATP6*	8697G>A	p.Met57 (=)	Silent	Homoplasmy	NP					
*MT-ATP6*	8818C>T	p.Leu98 (=)	Silent	Heteroplasmy	rs878853097					
*MT-ATP6*	8856G>A	p.Ala110 (=)	Silent	Homoplasmy	NP					
*MT-ATP6*	9042C>T	p.His172 (=)	Silent	Homoplasmy	NP					
*MT-ATP6*	9101T>C	p.Ile192Thr	Missense	Homoplasmy	rs199476134					
*MT-ATP6*	9123G>A	p.Leu199 (=)	Silent	Homoplasmy	NP					
*MT-CO3*	9288A>G	p.Thr28Ala	Missense	Homoplasmy	NP	9221A>G	p.Ser5 (=)	Silent	Homoplasmy	NP
*MT-CO3*	9320C>T	p.His38 (=)	Silent	Homoplasmy	NP	9380G>A	p.Trp58 (=)	Silent	Homoplasmy	NP
*MT-CO3*	9347A>G	p.Leu47 (=)	Silent	Homoplasmy	NP	9477G>A	p.Val91Ile	Missense	Homoplasmy	rs2853825
*MT-CO3*	9371C>T	p.Tyr55 (=)	Silent	Heteroplasmy	NP	9587A>G	p.Leu127 (=)	Silent	Homoplasmy	NP
*MT-CO3*	9419C>T	p.His71 (=)	Silent	Homoplasy	NP	9667A>G	p.Asn154Ser	Missense	Homoplasmy	rs41482146
*MT-CO3*	9494A>G	p.Gly96 (=)	Silent	Homoplasmy	NP					
*MT-CO3*	9755G>A	p.Glu183 (=)	Silent	Homoplasmy	NP					
*MT-CO3*	9818C>T	p.His204 (=)	Silent	Homoplasmy	NP					
*MT-CO3*	9932G>A	p.Trp242 (=)	Silent	Homoplasmy	NP					
*MT-ND3*	10237T>C	p.Ile60Thr	Missense	Heteroplasmy	rs1556423787					
*MT-ND3*	10373G>A	p.Glu105 (=)	Silent	Homoplasmy	NP	10115T>C	p.Ile19 (=)	Silent	Homoplasmy	NP
*MT-ND3*				Homoplasmy		10238T>C	p.Ile60 (=)	Silent	Homoplasmy	NP
*MT-ND4L*	10664C>T	p.Val65 (=)	Silent	Homoplasmy	NP					
*MT-ND4L*	10688G>A	p.Val73 (=)	Silent	Homoplasmy	rs2853488					
*MT-ND4*	10810T>C	p.Leu17 (=)	Silent	Homoplasmy	NP	11119C>T	p.Ile120 (=)	Silent	Heteroplasmy	NP
*MT-ND4*	10819A>G	p.Lys20 (=)	Silent	Heteroplasmy	NP	11207C>T	p.Leu150 (=)	Silent	Homoplasmy	NP
*MT-ND4*	10876A>G	p.Leu39 (=)	Silent	Homoplasmy	NP	11253T>C	p.Ile165Thr	Missense	Homoplasmy	NP
*MT-ND4*	10915T>C	p.Cys52 (=)	Silent	Homoplasmy	NP	11569T>C	p.Ile270 (=)	Silent	Homoplasmy	NP
*MT-ND4*	11176G>A	p.Gln139 (=)	Silent	Homoplasmy	NP	11674C>T	p.Thr305 (=)	Silent	Homoplasmy	NP
*MT-ND4*	11332C>T	p.Ala191 (=)	Silent	Heteroplasmy	NP	11797A>G	p.Gln346 (=)	Silent	Homoplasmy	NP
*MT-ND4*	11641A>G	p.Met294 (=)	Silent	Homoplasmy	NP	11815C>A	p.Leu352 (=)	Silent	Homoplasmy	rs879025367
*MT-ND4*	11761C>T	p.Tyr334 (=)	Silent	Homoplasmy	NP	11944T>C	p.Leu395 (=)	Silent	Homoplasmy	NP
*MT-ND4*	12007G>A	p.Trp416 (=)	Silent	Homoplasmy	NP	11947A>G	p.Thr396 (=)	Silent	Homoplasmy	NP
*MT-ND4*	12031G>A	p.Asn424Lys	Missense	Homoplasmy	NP					
*MT-ND4*	12084C>T	p.Ser442Phe	Missense	Homoplasmy	rs1556424051					
*MT-ND5*	12358A>G	p.Thr8Ala	Missense	Homoplasmy	rs201027657	12411C>T	p.Asn25 (=)	Silent	Homoplasmy	NP
*MT-ND5*	12530A>G	p.Asn65Ser	Missense	Homoplasmy	rs1603223785	12414T>C	p.Pro26 (=)	Silent	Homoplasmy	NP
*MT-ND5*	12615A>G	p.Ala93 (=)	Silent	Homoplasmy	NP	12501G>A	p.Met55 (=)	Silent	Homoplasmy	NP
*MT-ND5*	12633C>T	p.Ser99 (=)	Silent	Homoplasmy	NP	12693A>G	p.Lys119 (=)	Silent	Homoplasmy	NP
*MT-ND5*	12720A>G	p.Met128 (=)	Silent	Homoplasmy	NP	12715A>G	p.Thr127Ala	Missense	Homoplasmy	rs1603223875
*MT-ND5*	12753A>G	p.Gln139 (=)	Silent	Heteroplasmy	NP	12738T>G	p.Ala134 (=)	Silent	Homoplasmy	NP
*MT-ND5*						12879T>C	p.Gly181 (=)	Silent	Homoplasmy	NP
*MT-ND5*	12771G>A	p.Glu145 (=)	Silent	Homoplasmy	NP	13287C>T	p.Ile317 (=)	Silent	Heteroplasmy	NP
*MT-ND5*	13020T>C	p.Gly228 (=)	Silent	Homoplasmy	NP	13326T>C	p.Cys330 (=)	Silent	Homoplasmy	NP
*MT-ND5*	13105A>G	p.Ile257Val	Missense	Homoplasmy	rs2853501	13419A>G	p.Gly361 (=)	Silent	Homoplasmy	NP
*MT-ND5*	13276A>G	p.Met314Val	Missense	Homoplasmy	NP	13470A>G	p.Leu378 (=)	Silent	Homoplasmy	NP
*MT-ND5*	13368G>A	p.Gly344 (=)	Silent	Homoplasmy	NP	13485A>G	p.Met383 (=)	Silent	Heteroplasmy	rs28359176
*MT-ND5*	13392T>C	p.Asn352 (=)	Silent	Homoplasmy	NP	13590G>A	p.Leu418 (=)	Silent	Homoplasmy	NP
*MT-ND5*	13506C>T	p.Tyr390 (=)	Silent	Homoplasmy	NP	13651A>G	p.Thr439Ala	Missense	Homoplasmy	rs1569484594
*MT-ND5*	13588C>T	p.Leu418 (=)	Silent	Homoplasmy	NP	13617T>C	p.Ile427 (=)	Silent	Homoplasmy	NP
*MT-ND5*	13734T>C	p.Phe466 (=)	Silent	Homoplasmy	NP	13681A>G	p.Thr449Ala	Missense	Homoplasmy	rs386829187
*MT-ND5*	14094T>C	p.Leu586 (=)	Silent	Homoplasmy	NP	13803A>G	p.Thr489 (=)	Silent	Homoplasmy	NA
*MT-ND5*						13810G>A	p.Ala492Thr	Missense	Homoplasmy	rs1603224361
*MT-ND5*						14088T>C	p.Ile584 (=)	Silent	Homoplasmy	NP
*MT-ND5*						14106T>C	p.Ser590 (=)	Silent	Homoplasmy	NP
*MT-ND6*	14155C>T	p.Gly173 (=)	Silent	Heteroplasmy	NP	14179A>G	p.Tyr165 (=)	Silent	Heteroplasmy	NP
*MT-ND6*	14161A>G	p.Ala171 (=)	Silent	Heteroplasmy	NP	14410G>A	p.Val88 (=)	Silent	Homoplasmy	NP
*MT-ND6*	14178T>C	p.Ile166Val	Missense	homoplasmy	NP	14443C>T	p.Glu77 (=)	Silent	Homoplasmy	NP
*MT-ND6*	14305G>A	p.Ser123 (=)	Silent	Heteroplasmy	NP	14566A>G	p.Gly36 (=)	Silent	Homoplasmy	NP
*MT-ND6*	14308T>C	p.Gly122 (=)	Silent	Homoplasmy	NP	14633A>G	p.Met14Thr	Missense	Homoplasmy	rs1569484667
*MT-ND6*	14484T>C	p.Met64Val	Missense	Homoplasmy	rs199476104					
*MT-ND6*	14569G>A	p.Ser35 (=)	Silent	Homoplasmy	NP					
*MT-ND6*	14620C>T	p.Gly18 (=)	Silent	Heteroplasmy	NP					
*MT-CYB*	14818A>G	p.Pro24 (=)	Silent	Heteroplasmy	NP	14793A>G	p.His16Arg	Missense	Homoplasmy	rs2853504
*MT-CYB*	14905G>A	p.Met53 (=)	Silent	Homoplasmy	rs193302983	14890A>G	p.Gly48 (=)	Silent	Homoplasmy	NP
*MT-CYB*	15136C>T	p.Gly130 (=)	Silent	Homoplasmy	NP	14927A>G	p.Thr61Ala	Missense	Homoplasmy	rs201551481
*MT-CYB*	15310T>C	p.Ile188 (=)	Silent	Homoplasmy	NP	14971T>C	p.Tyr75 (=)	Silent	Homoplasmy	NP
*MT-CYB*	15388T>C	p.His214 (=)	Silent	Hetero	NP	15043G>A	p.Gly99 (=)	Silent	Homoplasmy	rs193302985
*MT-CYB*	15431G>A	p.Ala229Thr	Missense	Homoplasmy	rs193302993	15218A>G	p.Thr158Ala	Missense	Homoplasmy	NP
*MT-CYB*	15607A>G	p.Lys287 (=)	Silent	Homoplasmy	NP	15523C>T	p.Ala259 (=)	Silent	Homoplasmy	NP
*MT-CYB*	15637C>T	p.Ser297 (=)	Silent	Homoplasmy	rs527236190	15466G>A	p.Met240 (=)	Silent	Homoplasmy	NP
*MT-CYB*	15661C>T	p.Pro305 (=)	Silent	Homoplasmy	NP	15778C>T	p.Ser344 (=)	Silent	Homoplasmy	NP
*MT-CYB*	15693T>C	p.Met316Thr	Missense	Heteroplasmy	rs200975632	15784T>C	p.Pro346 (=)	Silent	Homoplasmy	rs527236194
*MT-CYB*	15787T>C	p.Phe347 (=)	Silent	Homoplasmy	NP					
*MT-CYB*	15850T>G	p.Thr368 (=)	Silent	Homoplasmy	NP					
*MT-CYB*	15884G>C	p.Ala380Pro	Missense	Heteroplasmy	rs527236195					

RRMS, relapse-remitting multiple sclerosis; NP, not provided.

Moreover, a large number of variants were found to be shared among patients and controls (Table 3). The majority of the observed variants showed no significant differences in their prevalence between patients and controls (P>0.05). Whereas the prevalence of 8 variants differed significantly between the two groups (P<0.05). Of these, two synonymous variants namely 152T>C and 263A>G (referred as rs285351 polymorphism) were located in the D-loop region of the mtDNA. The variant 152T>C was detected in 23% of patients and 13% of controls (P = 0.024). Whereas the variant 263A>G was found in 100% of patients and 50% of controls (P<0.01). Other 6 variants were found in protein-coding genes: The variant 4216T>C represented a non-synonymous polymorphism referred as rs1599988 in *MT-ND1* gene and was observed in 39% of patients compared to 13% of controls (P = 0.048). The variant 7028C>T represented a synonymous polymorphism referred as rs2015062 in *MT-CO1* gene and was observed in 96% of patients compared to 42% of controls (P<0.01). The variant 10398A>G represented a non-synonymous polymorphism referred as rs2853826 in *MT-ND3* gene and was found in 30% of patients and 63% of controls (P = 0.041). The variant 11719G>A represented a synonymous polymorphism in *MT-ND4* gene and was found in 83% of patients and 42% of controls (P = 0.006). The variant 13708G>A represented a non-synonymous polymorphism referred as rs28359178 in *MT-ND5* gene was detected in 39% of patients compared to 8% of controls (P = 0.017). Finally, the variant 14766C>T represented a non-synonymous polymorphisms referred as rs193302980 in *MT-CYB* gene and was observed in 96% of patients and 50% of controls (P = 0.003).

**Table 3:** Common mtDNA variants and their prevalence among RRMS patients and healthy controls.

Location	Nucleotide change	Amino acid change	Type of mutation	Nature	Variant ID	Patient’s frequency (%)	Control’s frequency (%)	OR	RR	95% CI	P value
D-loop	57insC	-	Insertion	Homoplasmy	NP	4	4	1	1	0.2–4	> 0.05
D-loop	64C>T	-	Substitution	Heteroplasmy	NP	17	13	1.3	1.3	0.6–2.5	> 0.05
D-loop	73A>G	-	Substitution	Homoplasmy	NP	74	75	0.94	0.98	0.8–1	> 0.05
D-loop	93A>G	-	Substitution	Homoplasmy	NP	4	4	1	1	0.2–4	> 0.05
D-loop	131T>C	-	Substitution	Homoplasmy	NP	4	8	0.4	0.5	0.15–1.6	> 0.05
D-loop	146T>C	-	Substitution	Homoplasmy	NP	17	21	0.7	0.8	0.4–1.4	> 0.05
D-loop	150C>T	-	Substitution	Homoplasmy	NP	17	13	1.3	1.3	0.6–2.5	> 0.05
D-loop	151C>T	-	Substitution	Homoplasmy	NP	4	4	1	1	0.2–4	> 0.05
**D-loop**	**152T>C**	**-**	**Substitution**	**Homoplasmy**	**NP**	**23**	**13**	**1.99**	**1.7**	**0.95–3.2**	**0.024**
D-loop	195T>C	-	Substitution	Homoplasmy	NP	35	33	1	1	0.7–1.5	> 0.05
D-loop	204T>C	-	Substitution	Heteroplasmy	NP	4	8	0.4	0.5	0.15–1.6	> 0.05
D-loop	207G>A	-	Substitution	Heteroplasmy	NP	4	8	0.4	0.5	0.15–1.6	> 0.05
D-loop	225G>A	-	Substitution	Heteroplasmy	NP	4	4	1	1	0.2–4	> 0.05
D-loop	235A>G	-	Substitution	Homoplasmy	NP	13	8	1.7	1.6	0.7–3.7	> 0.05
**D-loop**	**263A>G**	**-**	**Substitution**	**Homoplasmy**	**rs285351**	**100**	**50**	**99**	**1.98**	**1.6–2.4**	**<0.01**
D-loop	295C>T	-	Substitution	Homoplasmy	NP	35	42	0.7	0.8	0.5–1.1	> 0.05
D-loop	302insAC	-	Insertion	Homoplasmy	NP	70	71	0.9	0.9	0.8–1.1	> 0.05
D-loop	310insC	-	Insertion	Homoplasmy	NP	100	100	1	1	0.9–1	> 0.05
D-loop	462C>T	-	Substitution	Homoplasmy	NP	22	25	0.8	0.8	0.5–1	> 0.05
D-loop	489T>C	-	Substitution	Homoplasmy	NP	35	38	0.8	0.9	0.6–1.3	> 0.05
D-loop	508A>G	-	Substitution	Homoplasmy	NA	4	13	0.2	0.3	0.1–0.9	> 0.05
D-loop	513delCA	-	Deletion	Homoplasmy	NP	17	21	0.7	0.8	0.4–1.4	> 0.05
D-loop	16051A>G	-	Substitution	Homoplasmy	NP	22	4	6.7	5.5	2–15	> 0.05
D-loop	16069C>T	-	Substitution	Homoplasmy	NP	35	33	1	1	0.7–1.5	> 0.05
D-loop	16145G>A	-	Substitution	Homoplasmy	NP	17	13	1.3	1.3	0.6–2.5	> 0.05
D-loop	16172C>T	-	Substitution	Homoplasmy	NP	4	13	0.2	0.3	0.1–0.9	> 0.05
D-loop	16179delA	-	Deletion	Heteroplasmy	NP	13	8	1.7	1.6	0.7–3.7	> 0.05
D-loop	16182insC	-	Insertion	Heteroplasmy	NP	13	13	1	1	0.4–2	> 0.05
D-loop	16188C>T	-	Substitution	Homoplasmy	NP	17	17	1	1	0.5–1.8	> 0.05
D-loop	16189T>C	-	Substitution	Heteroplasmy	NP	30	25	1.2	1.2	0.7–1.8	> 0.05
D-loop	16126T>C	-	Substitution	Homoplasmy	NP	57	50	1	1	0.8–1.4	> 0.05
D-loop	16129G>A	-	Substitution	Homoplasmy	NP	9	4	2	2	0.7–7	> 0.05
D-loop	16222C>T	-	Substitution	Homoplasmy	NP	9	13	0.6	0.6	0.3–1.5	> 0.05
D-loop	16223C>T	-	Substitution	Homoplasmy	rs2853513	17	21	0.7	0.8	0.4–1.4	> 0.05
D-loop	16224T>C	-	Substitution	Homoplasmy	NP	4	17	0.2	0.2	0.08–0.6	> 0.05
D-loop	16243T>C	-	Substitution	Homoplasmy	NP	1	2	0.5	0.5	0.04–5	> 0.05
D-loop	16261C>T	-	Substitution	Homoplasmy	NP	17	8	2	2	0.9–4	> 0.05
D-loop	16278C>T	-	Substitution	Homoplasmy	NP	13	21	0.5	0.6	0.3–1	> 0.05
D-loop	16294C>T	-	Substitution	Homoplasmy	NP	4	4	1	1	0.2–4	> 0.05
D-loop	16300A>G	-	Substitution	Homoplasmy	NP	4	8	0.4	0.5	0.15–1.6	> 0.05
D-loop	16304T>C	-	Substitution	Homoplasmy	NP	4	4	1	1	0.2–4	> 0.05
D-loop	16311T>C	-	Substitution	Homoplasmy	NP	30	21	1.6	1.4	0.8–2	> 0.05
D-loop	16344C>T	-	Substitution	Heteroplasmy	NP	4	4	1	1	0.2–4	> 0.05
D-loop	16362T>C	-	Substitution	Homoplasmy	NP	26	25	1	1	0.6–1.6	> 0.05
D-loop	16355C>T	-	Substitution	Heteroplasmy	NP	17	4	4.9	4	1–12	> 0.05
D-loop	16399A>G	-	Substitution	Heteroplasmy	NP	4	4	1	1	0.2–4	> 0.05
D-loop	16519T>C	-	Substitution	Homoplasmy	NP	43	46	0.8	0.9	0.6–1	> 0.05
rRNA genes											
*MT-RNR1*	709G>A	-	Substitution	Heteroplasmy	NP	4	21	0.15	0.19	0.06–0.5	> 0.05
*MT-RNR1*	750A>G	-	Substitution	Homoplasmy	rs2853518	100	100	1	1	0.9–1	> 0.05
*MT-RNR1*	769G>A	-	Substitution	Homoplasmy	NP	4	4	1	1	0.2–4	> 0.05
*MT-RNR1*	1189T>C	-	Substitution	Homoplasmy		4	13	0.2	0.3	0.1–0.9	> 0.05
*MT-RNR1*	1438A>G	-	Substitution	Homoplasmy	rs2001030	100	100	1	1	0.9–1	> 0.05
*MT-RNR1*	1018G>A	-	Substitution	Homoplasmy	NP	4	8	0.4	0.5	0.15–1.6	> 0.05
*MT-RNR2*	1719G>A	-	Substitution	Homoplasmy	NP	17	8	2	2	0.9–4	> 0.05
*MT-RNR2*	1733C>T	-	Substitution	Homoplasmy	NP	13	8	1.7	1.6	0.7–3.7	> 0.05
*MT-RNR2*	1811A>G	-	Substitution	Heteroplasmy	NP	26	13	2	2	1–3	> 0.05
*MT-RNR2*	2355A>G	-	Substitution	Homoplasmy	NP	4	13	0.2	0.3	0.1–0.9	> 0.05
*MT-RNR2*	2442T>C	-	Substitution	Heteroplasmy	NP	17	13	1.3	1.3	0.6–2.5	> 0.05
*MT-RNR2*	2706A>G	-	Substitution	Homoplasmy	NP	96	88	3	1	1–1.1	> 0.05
*MT-RNR2*	2758G>A	-	Substitution	Homoplasmy	NP	4	4	1	1	0.2–4	> 0.05
*MT-RNR2*	3010G>A	-	Substitution	Homoplasmy		17	21	0.7	0.8	0.4–1.4	> 0.05
tRNA genes											
*MT-TQ* tRNA-Gln	4340A>G	-	Substitution	Homoplasmy	NP	4	4	1	1	0.2–4	> 0.05
*MT-TS1* tRNA-Ser	7476C>T	-	Substitution	Homoplasmy	rs201950015	9	13	0.6	0.6	0.3–1.5	> 0.05
*MT-TD* tRNA-Asp	7521G>A	-	Substitution	Homoplasmy	NP	4	8	0.4	0.5	0.15–1.6	> 0.05
*MT-TH* tRNA-His	12171A>G	-	Substitution	Homoplasmy	rs1603223589	13	4	3	3	1–9	> 0.05
*MT-TL2* tRNA-Leu	12308A>G	-	Substitution	Homoplasmy	NP	26	24	1	1	0.6–1.7	> 0.05
Protein-coding genes											
*MT-ND1*	3480A>G	p.Lys58 (=)	Silent	Homoplasmy	NP	9	13	0.6	0.6	0.3–1.5	> 0.05
*MT-ND1*	3531G>A	p.Pro75 (=)	Silent	Homoplasmy	NP	4	4	1	1	0.2–4	> 0.05
*MT-ND1*	3537A>G	p.Leu77 (=)	Silent	Homoplasmy	NP	4	4	1	1	0.2–4	> 0.05
*MT-ND1*	3594C>T	p.Val96 (=)	Silent	Homoplasmy	NP	4	8	0.4	0.5	0.15–1.6	> 0.05
*MT-ND1*	3847T>C	p.Leu181 (=)	Silent	Heteroplasmy	NP	17	13	1.3	1.3	0.6–2.5	> 0.05
*MT-ND1*	4104A>G	p.Leu266 (=)	Silent	Homoplasmy	NP	4	8	0.4	0.5	0.15–1.6	> 0.05
***MT-ND1***	**4216T>C**	**p.Tyr304His**	**Missense**	**Homoplasmy**	**rs1599988**	**39**	**13**	**4.2**	**3**	**1.7–5.2**	**0.048**
*MT-ND2*	4769A>G	p.Met100 (=)	Silent	Homoplasmy	rs3021086	91	100	0.1	0.9	0.8–0.9	> 0.05
*MT-ND2*	5460G>A	p.Ala331Thr	Missense	Homoplasmy	NP	4	4	1	1	0.2–4	> 0.05
*MT-CO1*	5981T>C	p.Ala26 (=)	Silent	Homoplasmy	NP	4	4	1	1	0.2–4	> 0.05
*MT-CO1*	6221T>C	p.Pro106 (=)	Silent	Homoplasmy	NP	13	8	1.7	1.6	0.7–3.7	> 0.05
*MT-CO1*	6366G>A	p.Val155Ile	Missense	Homoplasmy	NP	4	4	1	1	0.2–4	> 0.05
*MT-CO1*	6371C>T	p.Ser156 (=)	Silent	Homoplasmy	NP	13	4	3	3	1–9	> 0.05
*MT-CO1*	6671T>C	p.His256 (=)	Silent	Homoplasmy	rs1978028	13	13	1	1	0.4–2	> 0.05
***MT-CO1***	**7028C>T**	**p.Ala375 (=)**	**Silent**	**Homoplasmy**	**rs2015062**	**96**	**42**	**33**	**2.2**	**1.8–2.8**	**<0.01**
*MT-CO1*	7256C>T	p.Asn451 (=)	Silent	Homoplasmy	NP	4	8	0.4	0.5	0.15–1.6	> 0.05
*MT-CO2*	8269G>A	p.Ter228Ter	Stop	Homoplasmy	NP	4	8	0.4	0.5	0.15–1.6	> 0.05
*MT-ATP8*	8386C>T	p.Thr7 (=)	Silent	Homoplasmy	NP	13	8	1.7	1.6	0.7–3.7	> 0.05
*MT-ATP8*	8389A>G	p.Val8 (=)	Silent	Homoplasmy	NP	4	4	1	1	0.2–4	> 0.05
*MT-ATP6*	8473T>C	p.Pro36 (=)	Silent	Homoplasmy	rs3020563	13	4	3	3	1–9	> 0.05
*MT-ATP6*	8701A>G	p.Thr59Ala	Missense	Homoplasmy	NP	9	8	1	1	0.4–2	> 0.05
*MT-ATP6*	8860A>G	p.Thr112Ala	Missense	Homoplasmy	NP	91	100	0.1	0.9	0.8–0.9	> 0.05
*MT-ATP6*	9055G>A	p.Ala177Thr	Missense	Homoplasmy	rs193303045	13	13	1	1	0.4–2	> 0.05
*MT-ATP6*	9103T>C	p.Phe193Leu	Missense	Homoplasmy	rs1603222077	13	4	3	3	1–9	> 0.05
*MT-CO3*	9540T>C	p.Leu112 (=)	Silent	Homoplasmy	rs2248727	9	8	1	1	0.4–2	> 0.05
*MT-CO3*	9698T>C	p.Leu164 (=)	Silent	Homoplasmy	NP	9	13	06	0.6	0.3–1.5	> 0.05
***MT-ND3***	**10398A>G**	**p.Thr114Ala**	**Missense**	**Homoplasmy**	**rs2853826**	**30**	**63**	**0.25**	**0.47**	**0.3–0.6**	**0.041**
*MT-ND4L*	10499A>G	p.Leu10 (=)	Silent	Homoplasmy	rs1057520074	13	13	1	1	0.4–2	> 0.05
*MT-ND4L*	10550A>G	p.Met27 (=)	Silent	Homoplasmy	NP	4	13	0.2	0.3	0.1–0.9	> 0.05
*MT-ND4L*	10589G>A	p.Leu40 (=)	Silent	Homoplasmy	NP	4	4	1	1	0.2–4	> 0.05
*MT-ND4*	10873T>C	p.Pro38 (=)	Silent	Homoplasmy	rs2857284	9	8	1	1	0.4–2	> 0.05
*MT-ND4*	11002A>G	p.Gln81 (=)	Silent	Homoplasmy	NP	13	13	1	1	0.4–2	> 0.05
*MT-ND4*	11251A>G	p.Leu164 (=)	Silent	Homoplasmy	NP	39	38	1	1	0.7–1	> 0.05
*MT-ND4*	11299T>C	p.Thr180 (=)	Silent	Homoplasmy	NP	4	13	0.2	0.3	0.1–0.9	> 0.05
*MT-ND4*	11377G>A	p.Lys206 (=)	Silent	Homoplasmy	NP	13	13	1	1	0.4–2	> 0.05
*MT-ND4*	11440G>A	p.Gly227 (=)	Silent	Homoplasmy	NP	13	4	3	3	1–9	> 0.05
*MT-ND4*	11467A>G	p.Leu236 (=)	Silent	Homoplasmy	rs2853493	26	25	1	1	0.6–1.6	> 0.05
***MT-ND4***	**11719G>A**	**p.Gly320 (=)**	**Silent**	**Homoplasmy**	**NP**	**83**	**42**	**6.7**	**1.9**	**1.5–2.5**	**0.006**
*MT-ND4*	11914G>A	p.Thr385 (=)	Silent	Homoplasmy	**NP**	4	4	1	1	0.2–4	> 0.05
*MT-ND5*	12372G>A	p.Leu12 (=)	Silent	Homoplasmy	rs2853499	26	25	1	1	0.6–1.6	> 0.05
*MT-ND5*	12570A>G	p.Leu78 (=)	Silent	Homoplasmy	NP	23	23	1	1	0.6–1.6	> 0.05
*MT-ND5*	12612A>G	p.Val92 (=)	Silent	Homoplasmy		30	38	0.6	0.7	0.5–1	> 0.05
*MT-ND5*	12705C>T	p.Ile123 (=)	Silent	Homoplasmy	NP	17	21	0.7	0.8	0.4–1.4	> 0.05
*MT-ND5*	13188C>T	p.Thr284 (=)	Silent	Heteroplasmy	NP	17	13	1.3	1.3	0.6–2.5	> 0.05
*MT-ND5*	13650C>T	p.Pro438 (=)	Silent	Homoplasmy	rs2854123	4	8	0.4	0.5	0.15–1.6	> 0.05
***MT-ND5***	**13708G>A**	**p.Ala458Thr**	**Missense**	**Homoplasmy**	**rs28359178**	**39**	**8**	**7.3**	**4.8**	**2.4–9.9**	**0.017**
*MT-ND5*	13813G>A	p.Val493Ile	Missense	Homoplasmy	rs1556424332	4	3	1	1	0.3–5	> 0.05
*MT-ND5*	13966A>G	p.Thr544Ala	Missense	Homoplasmy	rs41535848	13	4	3	3	1–9	> 0.05
*MT-ND5*	14040G>A	p.Gln568 (=)	Silent	Homoplasmy	rs57180882	4	4	1	1	0.2–4	> 0.05
*MT-ND6*	14167C>T	p.Glu169 (=)	Silent	Homoplasmy	NP	9	13	0.6	0.6	0.3–1.5	> 0.05
*MT-ND6*	14470T>C	p.Gly68 (=)	Silent	Homoplasmy	rs3135030	13	4	3	3	1–9	> 0.05
*MT-ND6*	14544G>A	p.Leu44 (=)	Silent	Homoplasmy	NP	4	4	1	1	0.2–4	> 0.05
***MT-CYB***	**14766C>T**	**p.Thr7Ile**	**Missense**	**Homoplasmy**	**rs193302980**	**96**	**50**	**24**	**1.92**	**1.5–2.3**	**0.003**
*MT-CYB*	14798T>C	p.Phe18Leu	Missense	Homoplasmy	rs28357681	4	21	0.15	0.19	0.06–0.5	0.12
*MT-CYB*	14831G>A	p.Ala29Thr	Missense	Homoplasmy		4	4	1	1	0.2–4	> 0.05
*MT-CYB*	15257G>A	p.Asp171Asn	Missense	Homoplasmy	rs41518645	13	13	1	1	0.4–2	> 0.05
*MT-CYB*	15301G>A	p.Leu185 (=)	Silent	Homoplasmy	NP	4	8	0.4	0.5	0.15–1.6	> 0.05
*MT-CYB*	15326A>G	p.Thr194Ala	Missense	Homoplasmy	rs2853508	100	100	1	1	0.9–1	> 0.05
*MT-CYB*	15452C>A	p.Leu236Ile	Missense	Homoplasmy	rs193302994	39	38	1	1	0.7–1.4	> 0.05
*MT-CYB*	15674T>C	p.Ser310Pro	Missense	Homoplasmy	rs1603225419	4	13	0.2	0.3	0.1–0.9	> 0.05
*MT-CYB*	15679A>G	p.Lys311 (=)	Silent	Homoplasmy	NP	4	13	0.2	0.3	0.1–0.9	> 0.05

RRMS, relapse-remitting multiple sclerosis; NP, not provided; OR, odds ratio; RR, risk ratio; CI, confidence interval.

### Nonsynonymous mtDNA variants identified only in patients

We next focused on nonsynonymous mtDNA variants that present only in MS patients as a possible genetic predisposition to disease. A total of 34 nonsynonymous variants categorized as missense mutations were found only present in patients but not in controls, some were detected in one patient and others were found in more than one patient (Table 4). They were located within protein-coding genes, therefore potentially functional. Of these, the 12031G>A mutation in *MT-ND4* gene was never reported in the MTOMAP or other databases and thus classified as a novel mutation. While 2 mutations, namely 3865A>G in *MT-ND1* gene and 3944T>C in *MT-ND1* gene were previously reported in MS. The remaining 31 mutations were previously described to be associated with diseases including mitochondrial disorders, neurological disorders and other diseases (Table 4).

**Table 4:** Nonsynonymous mtDNA variants identified only in RRMS patients.

Patient ID	Gene	Nucleotide change	Amino acid change	Type of mutation	Nature	* Reported / Novel
P1	*MT-ND2*	4695T>C	p.Phe76Leu	Missense	Homoplasmy	Leigh syndrome
P2	*MT-ND2*	5073A>G	p.Ile202Val	Missense	Homoplasmy	Leigh syndrome
P2	*MT-ND2*	5442T>C	p.Phe325Leu	Missense	Homoplasmy	Mitochondrial disorders, Leigh syndrome, normal-tension glaucoma
P2	*MT-ND3*	10237T>C	p.Ile60Thr	Missense	Heteroplasmy	Mitochondrial disorder, Leigh syndrome
P2	*MT-CO1*	7146A>G	p.Thr415Ala	Missense	Homoplasmy	Leigh syndrome
P2	*MT-ATP6*	8566A>G	p.Ile14Val	Missense	Homoplasmy	Leigh syndrome
P2	*MT-CO3*	9288A>G	p.Thr28Ala	Missense	Homoplasmy	Leigh syndrome
P2	*MT-ND4*	12031G>A	p.Asn424Lys	Missense	Homoplasmy	Novel
P2	*MT-ND5*	13105A>G	p.Ile257Val	Missense	Homoplasmy	Mitochondrial disorder, Leigh syndrome, hearing-impairment
P2	*MT-ND5*	13276A>G	p.Met314Val	Missense	Homoplasmy	Leigh syndrome
P2	*MT-ND6*	14484T>C	p.Met64Val	Missense	Homoplasmy	Mitochondrial disorders
P2	*MT-CYB*	15431G>A	p.Ala229Thr	Missense	Homoplasmy	Leigh syndrome, cardiomyopathy, neoplasm of ovary
P4	*MT-ATP8*	8490T>C	p.Met42Thr	Missense	Homoplasmy	Leigh syndrome
P4	*MT-ND5*	12530A>G	p.Asn65Ser	Missense	Homoplasmy	Leigh syndrome
P4	*MT-ND6*	14178T>C	p.Ile166Val	Missense	Homoplasmy	Leigh syndrome, Neoplasm of ovary, dilated cardiomyopathy
P4, P12, P18	*MT-CYB*	15884G>C	p.Ala380Pro	Missense	Heteroplasmy	Mitochondrial disorder, Leigh syndrome, dilated cardiomyopathy,
P5, P20	*MT-ND1*	3865A>G	p.Ile187Val	Missense	Heteroplasmy	MS, Leigh syndrome
P10, P-11	*MT-ND4*	12084C>T	p.Ser442Phe	Missense	Homoplasmy	Parkinson disease
P13	*MT-ND1*	3944T>C	p.Ile213Thr	Missense	Heteroplasmy	MS
P14, P23	*MT-ND2*	5437C>T	p.Thr323Ile	Missense	Homoplasmy	Leigh syndrome
P14, P23	*MT-CYB*	15693T>C	p.Met316Thr	Missense	Heteroplasmy	Dementia, Leigh syndrome
P15	*MT-ATP6*	9101T>C	p.Ile192Thr	Missense	Homoplasmy	Mitochondrial disorders
P15	*MT-ND5*	12358A>G	p.Thr8Ala	Missense	Homoplasmy	Normal-tension glaucoma, metabolic syndrome
P16	*MT-ND2*	4917A>G	p.Asn150Asp	Missense	Homoplasmy	Leigh syndrome, Mitochondrial disorder, Parkinson disease
P18	*MT-ND1*	3316G>A	p.Ala4Thr	Missense	Homoplasmy	Mitochondrial disorder
P18	*MT-CO1*	6546C>T	p.Leu215Phe	Missense	Homoplasmy	Leigh syndrome
P22	*MT-ND1*	3533C>T	p.Thr76Ile	Missense	Homoplasmy	Leigh syndrome
P22	*MT-ND2*	5191C>T	p.Thr241Met	Missense	Homoplasmy	Leigh syndrome

RRMS, relapse-remitting multiple sclerosis; NP, not provided.

*Mutations were novel or known according to MITOMAP, mtDB, ClinVar databases and Google search.

### Prediction of impacts of missense mutations on protein function

To analyse the impacts of amino acid substitutions upon missense mutations on protein function, a combination of tools were used including PolyPhen, SIFT, CADD, and Mutation Assessor (Dong et al., 2015). Accordingly, the missense mutations were defined as deleterious when predicted to be damaging, probably/possibly damaging by these methods. The analysis predicted 7 mutations to be deleterious, all of which were not previously reported in MS (Table 5). Of these, 4 mutations namely 10237T>C in *MT-ND3* gene, 9288A>G in *MT-CO3* gene, 14484T>C in *MT-ND6* gene and 15431G>A in MT-CYB gene were all found in one patient (P2). Two mutations, namely 8490T>C in *MT-ATP8* gene and 15884G>C in *MT-CYB* gene were found in another patient (P4). The same mutation 15884G>C in *MT-CYB* gene was also found in two other patients (P12 and P18). Whereas, the mutation 5437C>T in *MT-ND2* gene was in two additional patients (P14 and P23).

**Table 5:** Prediction of impacts of missense mutations on protein function.

Patient ID	Gene	Nucleotide change	Amino acid change	Type of mutation	Nature	* Reported in MS	Prediction			
							PolyPhen	SIFT	CAAD	Mutation Assessor
P1	*MT-ND2*	4695T>C	p.Phe76Leu	Missense	Homoplasmy	No	Benign	Tolerated	Neutral	Low
P2	*MT-ND2*	5073A>G	p.Ile202Val	Missense	Homoplasmy	No	Benign	Tolerated	Neutral	Medium
P2	*MT-ND2*	5442T>C	p.Phe325Leu	Missense	Homoplasmy	No	Benign	Tolerated	Neutral	Low
**P2**	***MT-ND3***	**10237T>C**	**p.Ile60Thr**	**Missense**	**Heteroplasmy**	No	**Probably damaging**	**Damaging**	**Damaging**	**High**
P2	*MT-CO1*	7146A>G	p.Thr415Ala	Missense	Homoplasmy	No	Benign	Tolerated	Neutral	Medium
P2	*MT-ATP6*	8566A>G	p.Ile14Val	Missense	Homoplasmy	No	Benign	Tolerated	Neutral	Low
**P2**	***MT-CO3***	**9288A>G**	**p.Thr28Ala**	**Missense**	**Homoplasmy**	No	**Probably damaging**	**Damaging**	**Neutral**	**Medium**
P2	*MT-ND4*	12031G>A	p.Asn424Lys	Missense	Homoplasmy	No	Benign	Tolerated	Neutral	Low
P2	*MT-ND5*	13105A>G	p.Ile257Val	Missense	Homoplasmy	No	Benign	Tolerated	Neutral	Low
P2	*MT-ND5*	13276A>G	p.Met314Val	Missense	Homoplasmy	No	Benign	Tolerated	Neutral	Medium
**P2**	***MT-ND6***	**14484T>C**	**p.Met64Val**	**Missense**	**Homoplasmy**	No	**Probably damaging**	**Damaging**	**Damaging**	**High**
**P2**	***MT-CYB***	**15431G>A**	**p.Ala229Thr**	**Missense**	**Homoplasmy**	No	**Possibly damaging**	**Damaging**	**Neutral**	**Medium**
**P4**	***MT-ATP8***	**8490T>C**	**p.Met42Thr**	**Missense**	**Homoplasmy**	No	**Probably damaging**	**Damaging**	**Damaging**	**Medium**
P4	*MT-ND5*	12530A>G	p.Asn65Ser	Missense	Homoplasmy	No	Benign	Tolerated	Neutral	Medium
P4	*MT-ND6*	14178T>C	p.Ile166Val	Missense	Homoplasmy	No	Benign	Tolerated	Benign	Medium
**P4, P12, P18**	***MT-CYB***	**15884G>C**	**p.Ala380Pro**	**Missense**	**Heteroplasmy**	No	**Possibly damaging**	**Tolerated**	**Damaging**	**Medium**
P5, P20	*MT-ND1*	3865A>G	p.Ile187Val	Missense	Heteroplasmy	Yes	Benign	Tolerated	Neutral	Medium
P10, P11	*MT-ND4*	12084C>T	p.Ser442Phe	Missense	Homoplasmy	No	Benign	Tolerated	Neutral	Low
P13	*MT-ND1*	3944T>C	p.Ile213Thr	Missense	Heteroplasmy	Yes	Benign	Tolerated	Neutral	Moderate
**P14, P23**	***MT-ND2***	**5437C>T**	**p.Thr323Ile**	**Missense**	**Homoplasmy**	No	**Probably damaging**	**Damaging**	**Damaging**	**Medium**
P14, P23	*MT-CYB*	15693T>C	p.Met316Thr	Missense	Heteroplasmy	No	Benign	Tolerated	Neutral	Low
P15	*MT-ATP6*	9101T>C	p.Ile192Thr	Missense	Homoplasmy	No	Benign	Tolerated	Neutral	Low
P15	*MT-ND5*	12358A>G	p.Thr8Ala	Missense	Homoplasmy	No	Benign	Tolerated	Neutral	**Medium**
P16	*MT-ND2*	4917A>G	p.Asn150Asp	Missense	Homoplasmy	No	Benign	Tolerated	Neutral	Moderate
P18	*MT-ND1*	3316G>A	p.Ala4Thr	Missense	Homoplasmy	No	Benign	Tolerated	Neutral	Low
P18	*MT-CO1*	6546C>T	p.Leu215Phe	Missense	Homoplasmy	No	Benign	Tolerated	Neutral	Low
P22	*MT-ND1*	3533C>T	p.Thr76Ile	Missense	Homoplasmy	No	Benign	Tolerated	Neutral	Medium
P22	*MT-ND2*	5191C>T	p.Thr241Met	Missense	Homoplasmy	No	Benign	Tolerated	Neutral	Medium

Mutations were defined as deleterious by PolyPhen, SIFT, CADD, and Mutation Assessor when predicted to be damaging, probably/possibly damaging.

*Mutations were reported in MS according to MITOMAP, mtDB, ClinVar databases and Google search.

### Prediction of impacts of missense mutations on protein structure

To analyze the impacts of deleterious missense mutations on protein structure, we have further studied the 3-D structure of encoded proteins for investigating the structural changes caused by specific mutation. The PyMOL (Molecular Graphics System Version 2.0, Schrodinger LLC) was used and the 3-D protein structure was retrieved from https://www.ebi.ac.uk/pdbe. The PyMOL was used to induce the deleterious mutations observed in mtDNA-encoded genes of the OXPHOS system including *MT-ND2* and *MT-ND6* of Complex I, *MT-CO3* of Complex IV, and *MT-CYB* of Complex III of OXPHOS system on 3-D retrieved models: 5xtd, 5xtd, 5z62 and 5xte, respectively. Each mutation was examined at the level of polar bonds and clashes. However, the effect of mutations in *MT-ATP8* gene of Complex V could not be illustrated at the protein structural level as the human 3-D structure are not available for Complex V proteins.

The mutation 10237T>C of *MT-ND3* gene has change in amino acid at Ile60Thr (S1 Fig). The wildtype Ile60 (red) (A) forms three polar bonds (yellow dashed lines) with Phe56, Leu-63 and Leu-64 (orange). Whereas the mutated Thr60 (red) (B) forms five polar bonds (yellow dashed lines) with Phe56, Leu57, Thr61, Leu63 and Leu64 (orange), and causes physical clashes with the surrounding amino acids (C). The mutation 9288A>G of *MT-CO3* gene has change in amino acid at Thr28Ala (S2 Fig). The wild type Thr28 (red) (A) forms four polar bonds (yellow dashed lines) with Ala24, Leu25 and Ser29 (all in orange). Whereas the mutated Ala28 (red) (B) shows a total loss of polar bonds, and causes physical clashes with the surrounding amino acids (C). The mutation 14484T>C of *MT-ND6* gene has change in amino acid at Met64Val (S3 Fig). The wildtype Met64 (red) (A) does not form any polar bonds with the surrounding amino acids. Whereas the mutated Val64 (red) (B) forms a single polar bond with Gly-68 (orange) and causes physical clashes with the surrounding amino acids (red) (C). The mutation 15431G>A of *MT-CYB* gene has change in amino acid at Ala229Thr (S4 Fig). Both the wildtype Ala229 (red) (A) and the mutated Thr229 (red) (B) form two polar bonds (yellow dashed lines) with the surrounding amino acids (orange). However, the mutated Thr229 causes physical clashes with the surrounding amino acids (red) (C). For the mutation 15884G>C of *MT-CYB* that has change in amino acid at Ala380Pro, the 3-D structure could not be done. Although alanine is the last amino acid within the protein, it was not shown in the 3-D structure, whereas the last shown amino acid was tryptophan 379. The mutation 5437C>T of *MT-ND2* gene has change in amino acid at Thr323Ile (S5 Fig). The wildtype Thr323 (red) (A) forms a single polar bond (yellow dashed line) with Pro322 (orange), while the mutated Ile323 (red) (B) shows a total loss of polar bonds and causes physical clashes with the surrounding amino acids (C).

## Discussion

Mutations in the mtDNA and disruption of mitochondrial function alter brain energy metabolism and cause neurodegeneration [9, 10, 18]. Previous studies on MS have used specific primers for targeted regions of mtDNA [19–21] or investigated specific mitochondrial polymorphisms and haplogroups for disease association [22–25]. In this study, we analyzed the whole mitochondrial genome to examine mtDNA mutations/variants in blood samples from Saudi patients with RRMS and healthy controls using NGS. Overall we identified 3248 variants, 1686 in patients and 1562 in controls that were distributed around all regions of mitochondrial genome (Fig 1A). We observed a large number of variants located in the D-loop region with a higher number of variants in patients than in controls (Fig 1A). There was an excess of homoplasmic variants (85% in patients and 86% in controls) compared to heteroplasmic variants (15% in patients and 14% in controls) (Fig 1B). When we analyzed the variants only observed in patients or only observed in controls, we found distinct unique variants in patients or controls (Fig 2 and Table 2). There were 177 synonymous variants only present in patients versus 152 synonymous variants only present in the controls. While there were 141 nonsynonymous (34 missense and 107 silent) variants among patients versus 118 nonsynonymous (29 missense and 89 silent) variants only present in controls. We also found a large number of common synonymous and nonsynonymous variants that were shared among patients and controls (Table 3). Common genetic variants which frequently occur in the human mtDNA are expected to be under selective constraints [28]. Such variants have functional qualities and can disturb mitochondrial function since natural selection acts on phenotype [29]. Indeed, mtDNA variants can influence mitochondrial activities [30], mtDNA transcription [31] and generation of ROS [32]. The impact of mtDNA variants has been also implicated in aging and age-related diseases [33]. Because of the degeneracy of the genetic code in which more than one codon may specify the same amino acid, many mutations in coding sequences, especially at the third base of codons, do not affect protein sequence or structure and function of proteins, and are therefore considered silent. However, it has been shown that silent mutations created by synonymous DNA changes can have a significant impact on mRNA stability and folding, and consequently gene expression [34–37]. They also influence the rate of translation as well as posttranslational modification of proteins [31, 38].

Some of the common mtDNA variants in our study showed differences in their prevalence among patients and controls (P<0.05) (Table 3). In the D-loop region, the synonymous variants 152T>C and 263A>G occurred at significantly higher frequencies in patients (23% and 100% respectively) compared to controls (13% and 50% respectively). The mitochondrial non-coding D-loop is known to accumulate mutations at a higher rate than other regions of mtDNA. Variants in the D-loop region have important contribution to the proper functioning of mitochondria through an effect on mtDNA replication, transcription or translation [31]. While variants in the mtDNA D-loop have been described in diseases such as cancer [39], cardiovascular disease [40] and Huntington’s disease [41], both 152T>C and 263A>G variants found in this study were not previously reported in MS.

In protein-coding genes also differed significantly in their frequencies among patients and controls. Namely, 4216T>C in *MT-ND1* gene (referred as rs1599988), 13708G>A in *MT-ND5* gene (referred as rs28359178) and 14766C>T in *MT-CYB* gene (referred as rs193302980) are all missense mutations, observed more frequent in patients (39%, 39% and 96% respectively) than in controls (13%, 8% and 50% respectively). 4216T>C and 13708G>A were previously reported in MS patients and considered as diseases predisposing markers [42, 43]. Whereas 14766C>T variant was linked to 13708G>A variant, a potential marker associated with the increased risk of MS in European cohorts [43]. Other variants namely 7028C>T in *MT-CO1* gene (referred as rs2015062) and 11719G>A in *MT-ND4* gene are silent polymorphisms, found more frequently in patients (96 and 83% respectively) than in controls (40 and 42% respectively). Both 7028C>T and 11719G>A were reported in MS patients from different populations [21, 43]. Contrary to these results, 7028C>T was not found to be associated with MS in a Russian cohort [44]. The variability in the occurrence and prevalence of these variants in MS patients in different studies may be due to genetic and racial factors, which differ among populations. Nevertheless, we could not exclude the possibility that these variants may be possible risk factors for MS. In addition, 10398A>G (referred as rs2853826) in *MT-ND3* gene represented a missense mutation and was found less frequently in patients (30%) than in controls (63%). This mutation was reported in previous studies to reduce the risk to Parkinson’s disease [45, 46], and could have a similar effect in MS.

Analysis of nonsynonymous variants detected only in patients but not in controls (Table 4) revealed 34 missense mutations. According to MITOMAP, mtDB and ClinVar as well as Google search, one mutation namely 12031G>A in *MT-ND4* gene was assessed as a novel mutation since it was not found in the above mentioned data sets and was non haplogroup associated with Phylotree. Whereas 2 mutations namely 3865A>G in *MT-ND1* gene and 3944T>C in *MT-ND1* gene were previously reported in MS, and the 31 mutations were previously described in several diseases such as mitochondrial disorders and neurological diseases. Pathogenic variants in the mtDNA due to changes in the amino acid sequences of protein coding genes have been associated with susceptibility to complex diseases [47]. The substitution of amino acid may cause change in the hydrophobicity/ hydrophilicity and influence the charges or binding properties of a given protein, thus affects the protein structure and function [48]. The PolyPhen, SIFT, CADD, and Mutation Assessor which are widely used to predict the effects of nonsynonymous mutations on protein function [27], confirmed 7 deleterious missense mutations (Table 5). The mutations were identified in different mtDNA-encoded genes of the ETC complexes. Three of these mutations were found in NADH dehydrogenase subunits: The transition mutation 5437C>T in *MT-ND2* is homoplasmic and causes changes in amino acid from Thr323Ile. The transition mutation 10237T>C in *MT-ND3* gene is heteroplasmic and causes changes in amino acid from Ile60Thr. The transition mutation 14484T>C in *MT-ND6* gene is homoplasmic and causes changes in amino acid from Met64Val. Threonine (Thr) is a neutral uncharged polar amino acids, whereas isoleucine (Ile), methionine (Met) and valine (Val) are hydrophobic uncharged nonpolar. NADH dehydrogenase (complex I) is the first enzyme of the respiratory chain, which catalyzes the transfer of electrons from NADH to ubiquinone. It a multi-subunit complex in which 7 subunits (ND1, ND2, ND3, ND4, ND4L, ND5 and ND6) are encoded by the mtDNA genes. There is a causative connection between defects in complex I and neurodegenerative diseases such as Parkinson’s disease [49] and MS [50], and variants in mtDNA-encoded complex I genes are implicated in the pathogenicity or risk of MS [19–21]. The identified deleterious mutations in NADH dehydrogenase subunits are likely to impair the function of the whole enzyme as deduced from their effects on complex I.

Another deleterious mutation was found in Cytochrome c oxidase, namely 9288A>G in *MT-CO3* gene. This transition mutation is homoplasmic and causes changes in amino acid from Thr28Ala. Threonine (Thr) is neutral uncharged polar amino acids, whereas alanine (Ala) is hydrophobic uncharged nonpolar. Cytochrome c oxidase (complex IV) catalyzes the final step in mitochondrial ETC, functioning in the transfer of electrons from cytochrome c to oxygen that is then converted to water. It consists of multi-subunit essential for assembly and respiratory function of the enzyme complex [51]. Three of the biggest subunits (COX I, COX II and COX III) are encoded by the mtDNA and form the functional core of the enzyme complex. Mutations in mtDNA-encoded COX subunits can lead to complex IV deficiency by affecting complex structure and assembly [51]. Functional defects of mitochondrial COX have been reported in MS lesions [52] and our study is the first to report the harmful effect of 9288A>G mutation of *MT-CO3* gene in MS patients.

Three other mutations were found in *MT-CYB* gene: The transition mutation 15431G>A is homoplasmic and causes changes in amino acid from Ala229Thr. The transition mutation 15674T>C is also homoplasmic and causes changes in amino acid from Ser310Pro. Whereas the transversion mutation 15884G>C is heteroplasmic and causes changes in amino acid from Ala380Pro. Alanine (Ala) is a hydrophobic uncharged nonpolar amino acid, threonine (Thr) and serine (Ser) are neutral uncharged polar amino acids, and proline (Pro) is a neutral uncharged nonpolar amino acid. The *MT-CYB* gene is a component of the ubiquinol-cytochrome c reductase (cytochrome b-c1 complex or complex III), the third complex in the ETC which plays a critical role in ATP generation. It contributes to the generation of a proton gradient across the mitochondrial membrane that is then used for ATP synthesis. The deleterious mutations identified in *MT-CYB* gene may have an important impact on complex III, particularly the heteroplasmic transversion mutation 15884G>C. The guanine base (G) in genomic DNA is highly susceptible to oxidative stress due to having the lowest oxidation potential. Transversion mutations such as G>C frequently occur under oxidative stress [53], an important factor in the progression of MS [54] and other neurodegenerative diseases [55].

Additionally the mutation 8490T>C was found in *MT-ATP8* gene of mitochondrial ATP synthase of complex V. This transition mutation is homoplasmic and causes changes in amino acid from Met42Thr. Methionine (Met) is a hydrophobic uncharged nonpolar amino acid, whereas threonine (Thr) is a neutral uncharged amino acids. Mitochondrial ATP synthase composed of 17 subunits, of which 2 (ATP6 and ATP8) are encoded by the mtDNA. Complex V is responsible for last step of OXPHOS and synthesizes ATP from ADP in the mitochondrial matrix using the energy provided by the proton electrochemical gradient [56]. While the 8490T>C mutation in *MT-ATP8* gene was not previously reported in MS, different pathogenic mutations in MT-ATP8 gene were described in human disorders such as epilepsy, MIDD, brain pseudo-atrophy, episodic weakness and neurological disorders [57]. Mutations in *MT-ATP8* gene not only disturb the ATP synthase function but can also affect the assembly process and stability of complex V [57].

The structure of a protein is closely linked to its stability, function, and interaction. Mutations that predominantly impact the function or structure of proteins show their effects through several factors including impairment of the catalytic machinery, disruption of a functional site, modification of inter-subunit interfaces, disruption of protein folding and destabilization of the protein [58, 59]. In addition to the harmful effects of mtDNA missense mutations on protein function, these mutations disturbed the structure of encoded-proteins and cause physical clashes with the surrounding amino acids as indicated by PyMOL analysis (S1–S5 Figs). When the substituting residue does not fit in the protein structure as a result of clashes with the surrounding amino acids, a drastic conformation change occurs as the consequence, which leads to local or global structural alteration in the protein [60]. Moreover, clashes of amino acids can drastically alter the folding and stability of proteins [59]. Therefore, these mutations appear functional and structural impact on encoded-proteins.

In our study, the effect of the mutation 8490T>C (Met42Thr) in *MT-ATP8* gene could not be illustrated at the protein structural level. However as mentioned above, replacement of amino acid from methionine to threonine due to this mutation can change the polarity of amino acids and hence affect the function and structure of Complex V.

In light of all this information, it can be postulated that the identified deleterious missense mutations in MS patients may be disease-related since they are located in structurally/functionally important sites and could impair the encoded-proteins involved in the OXPHOS system in mitochondria.

The mtDNA is particularly susceptible to acquiring somatic mutations due to its close proximity to the ROS production site, lack of protective histone and insufficient repair systems [15]. The accumulation of mtDNA mutations impairs the ETC, triggers oxidative damage, and subsequently a decline in mitochondrial function with more ROS production. Mitochondrial dysfunction as a result of mtDNA mutations can lead to to energy failure, a hallmark of neurodegenerative disease and contributes to axonal degeneration in MS [10, 18, 55]. Given the important role of the ETC complexes, particularly complexes I and complex III in ROS generation [61, 62], the detected deleterious mutations in these complexes may be implicated in MS by affecting the mitochondrial ROS production. However, it is difficult to identify ROS-causing or -pathogenic mutations because of the co-occurrence of multiple mtDNA mutations in several mitochondrial complexes.

When we further analyzed the patient-specific mutations (Table 5), we observed that some patients harboured multiple mutations, while other patients carried the same mutations. In this respect, patient P2 carried four mutations; the heteroplasmic mutation 10237T>C in *MT-ND3* gene, and the homoplasmic mutations 9288A>G in *MT-CO3* gene, 1484T>C in *MT-ND6* gene and 15431G>A in *MT-CYB* gene. Patient P4 carried the homoplasmic mutation 8490T>C in *MT-ATP8* gene and the heteroplasmic mutation 15884G>C in *MT-CYB* gene. The same heteroplasmic mutation 15884G>C in *MT-CYB* gene was also carried by two other patients (P12 and P18). Whereas two patients (P14 and P23) carried the homoplasmic mutation 5437C>T in *MT-ND2* gene. Studying other family members of those patients, especially patient P2 who carried four harmful mutations which can lead to respiratory defects, is significant to further understand the role of mitochondrial neurogenetics in MS. Nevertheless, when mtDNA mutations are studied in a complex disease as MS, other modifying factors including environmental factors and the possible effects of the nuclear genes should be considered for the phenotypic manifestation of mutations.

## Conclusions

Taken together, out of a large number of variants, we found distinct unique variants that were only present in the patients or only occur in the controls. We also found a number of common variants and the prevalence of some of them differed significantly between patients and controls. Additionally we identified 34 missense mutations only present in the patients. Of them, seven mutations (including homoplasmic and heteroplasmic) were not previously reported in MS, and predicted to be deleterious with considerable impacts on the functions and structures of encoded-proteins. We also observed that some patients harbored multiple deleterious mutations while other patients carried the same deleterious mutations. While the identified mtDNA sequence variants are not sufficient to cause a classical disease, they may be associated with a cascade involving altered energy output, and thus could contribute to the susceptibility or pathogenicity of MS. To the best of our knowledge, this is the first study to perform sequence analysis of the whole mtDNA of MS patients in an Arab population. Our results expanded the mutational spectrum of mtDNA variants in MS and highlighted the efficiency of NGS in population-specific mtDNA variant discovery. A more extensive analysis using a larger sample size is required to confirm the link between mitochondrial neurogenetics and MS.

## Supporting information

S1 Fig 10237T>C (Ile60Thr) mutation of MT-ND3 gene. The wildtype Ile60 (red) (A) forms three polar bonds (yellow dashed lines) with Phe56, Leu-63 and Leu-64 (all in orange). Whereas the mutated Thr60 (red) (B) forms five polar bonds (yellow dashed lines) with Phe56, Leu57, Thr61, Leu63 and Leu64 (orange), and causes physical clashes with the surrounding amino acids (C).
(PDF)

S2 Fig 9288A>G (Thr28Ala) mutation of MT-CO3 gene. The wild type Thr28 (red) (A) forms four polar bonds (yellow dashed lines) with Ala24, Leu25 and Ser29 (all in orange). Whereas the mutated Ala28 (red) (B) shows a total loss of polar bonds, and causes physical clashes with the surrounding amino acids (C).
(PDF)

S3 Fig 14484T>C (Met64Val) mutation of MT-ND6 gene. The wildtype Met64 (red) (A) does not form any polar bonds with the surrounding amino acids. Whereas the mutated Val64 (red) (B) forms a single polar bond with Gly-68 (orange) and causes physical clashes with the surrounding amino acids (red) (C).
(PDF)

S4 Fig 15431G>A (Ala229Thr) mutation of MT-CYB gene. Both the wildtype Ala229 (red) (A) and the mutated Thr229 (red) (B) form two polar bonds (yellow dashed lines) with the surrounding amino acids (all in orange). However, the mutated Thr229 causes physical clashes with the surrounding amino acids (red) (C).
(PDF)

S5 Fig 5437C>T (Thr323Ile) mutation of MT-ND2 gene. The wildtype Thr323 (red) (A) forms a single polar bond (yellow dashed line) with Pro322 (orange), while the mutated Ile323 (red) (B) shows a total loss of polar bonds and causes physical clashes with the surrounding amino acids (C).
(PDF)
