# Supplementary figures and images for: Next-generation sequencing of the whole mitochondrial genome identifies functionally deleterious mutations in patients with multiple sclerosis
Source: PLoS One. 2022 Feb 7;17(2):e0263606. doi: 10.1371/journal.pone.0263606 (PMC8820615; doi:10.1371/journal.pone.0263606)

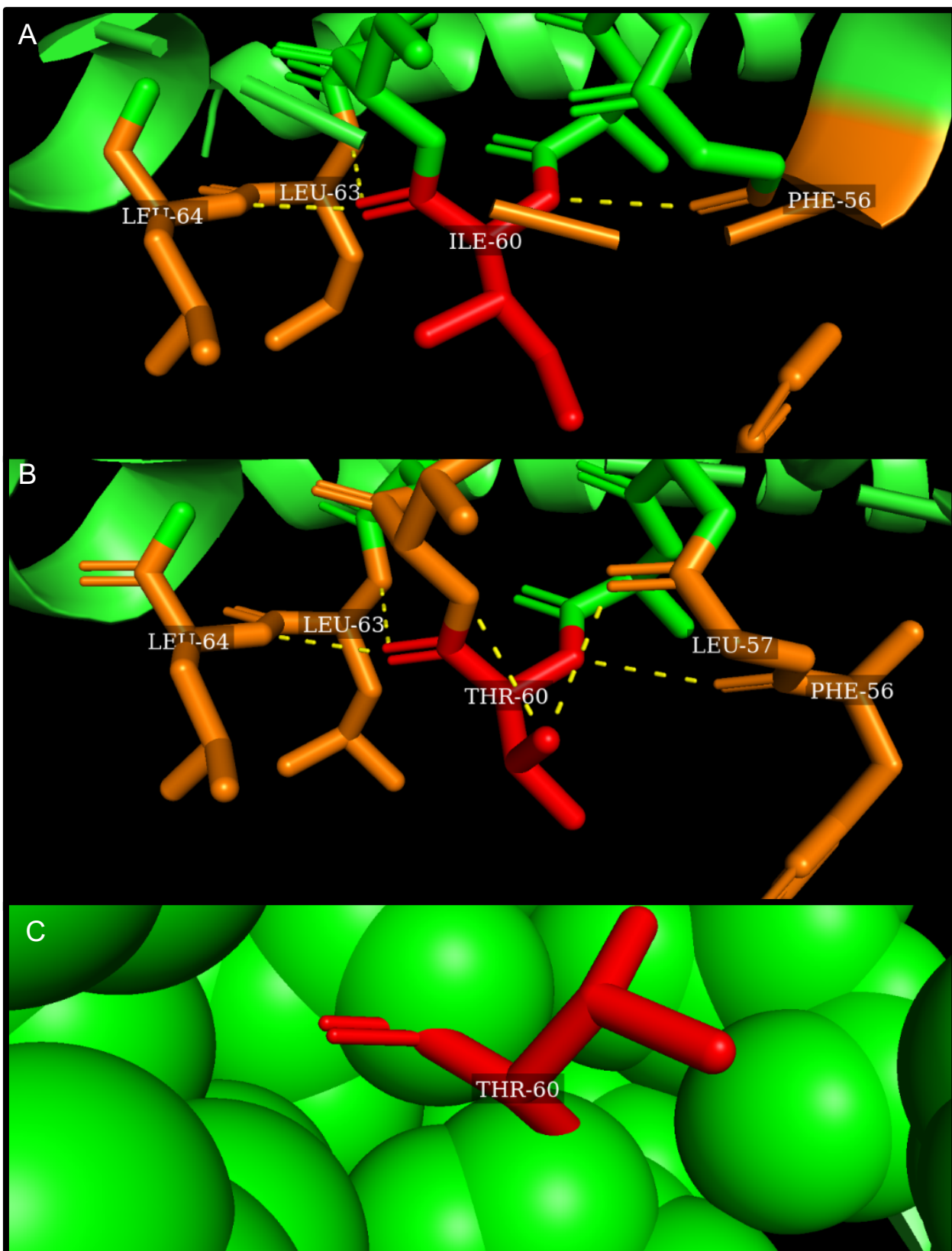

Supplement: S1 Fig — The wildtype Ile60 (red) (A) forms three polar bonds (yellow dashed lines) with Phe56, Leu-63 and Leu-64 (all in orange). Whereas the mutated Thr60 (red) (B) forms five polar bonds (yellow dashed lines) with Phe56, Leu57, Thr61, Leu63 and Leu64 (orange), and causes physical clashes with the surrounding amino acids (C). (PDF) [file pone.0263606.s001.pdf]

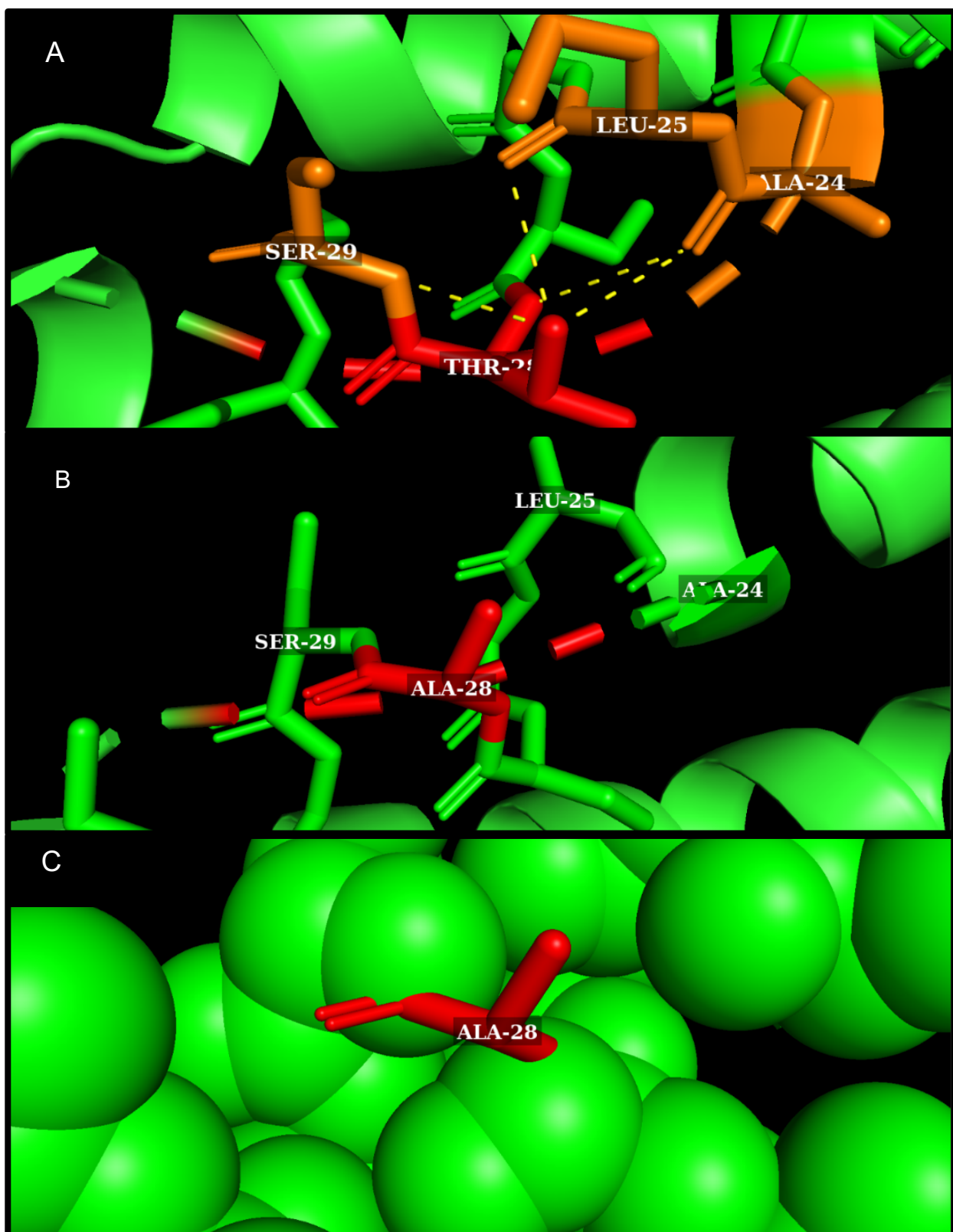

Supplement: S2 Fig — The wild type Thr28 (red) (A) forms four polar bonds (yellow dashed lines) with Ala24, Leu25 and Ser29 (all in orange). Whereas the mutated Ala28 (red) (B) shows a total loss of polar bonds, and causes physical clashes with the surrounding amino acids (C). (PDF) [file pone.0263606.s002.pdf]

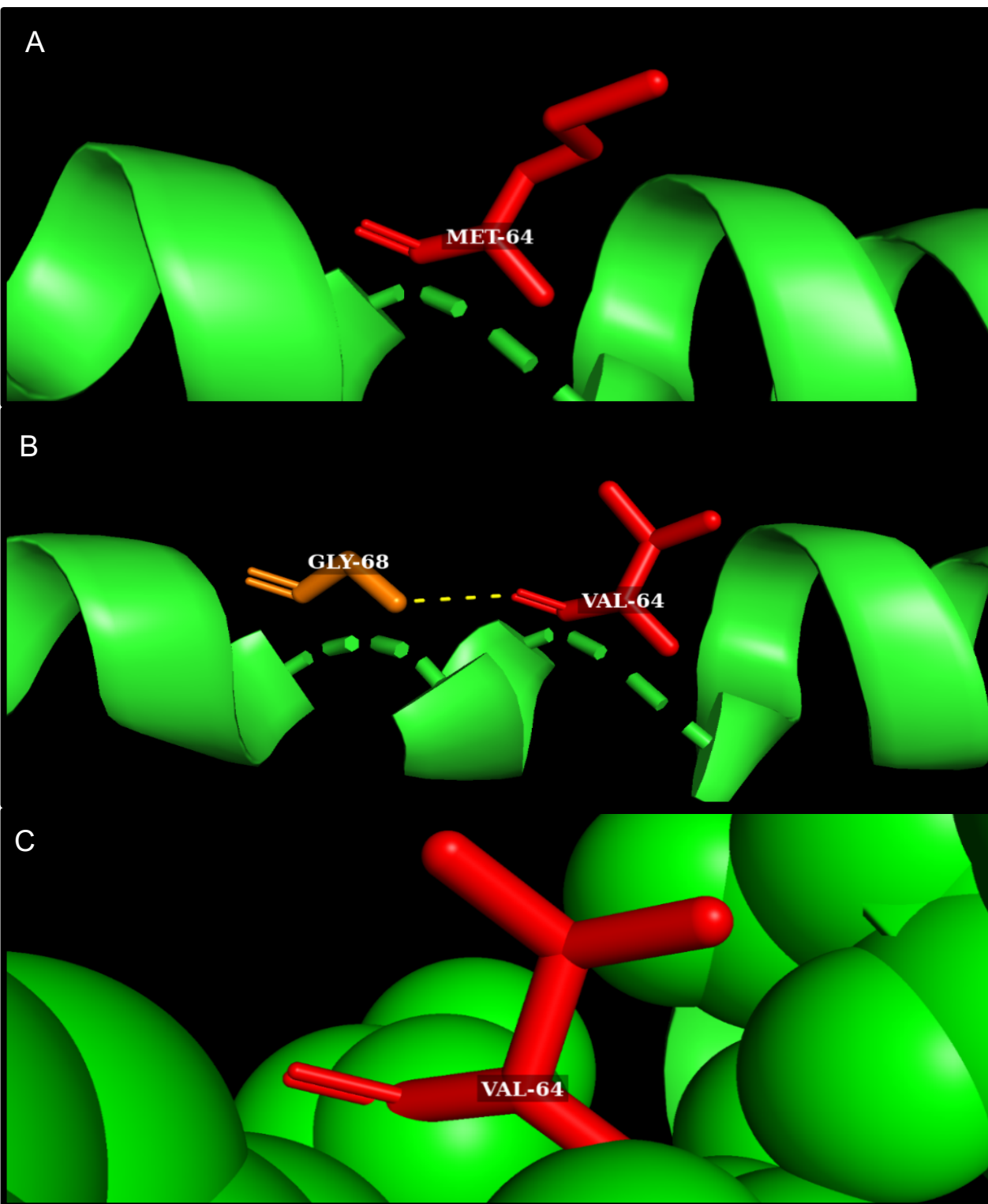

Supplement: S3 Fig — The wildtype Met64 (red) (A) does not form any polar bonds with the surrounding amino acids. Whereas the mutated Val64 (red) (B) forms a single polar bond with Gly-68 (orange) and causes physical clashes with the surrounding amino acids (red) (C). (PDF) [file pone.0263606.s003.pdf]

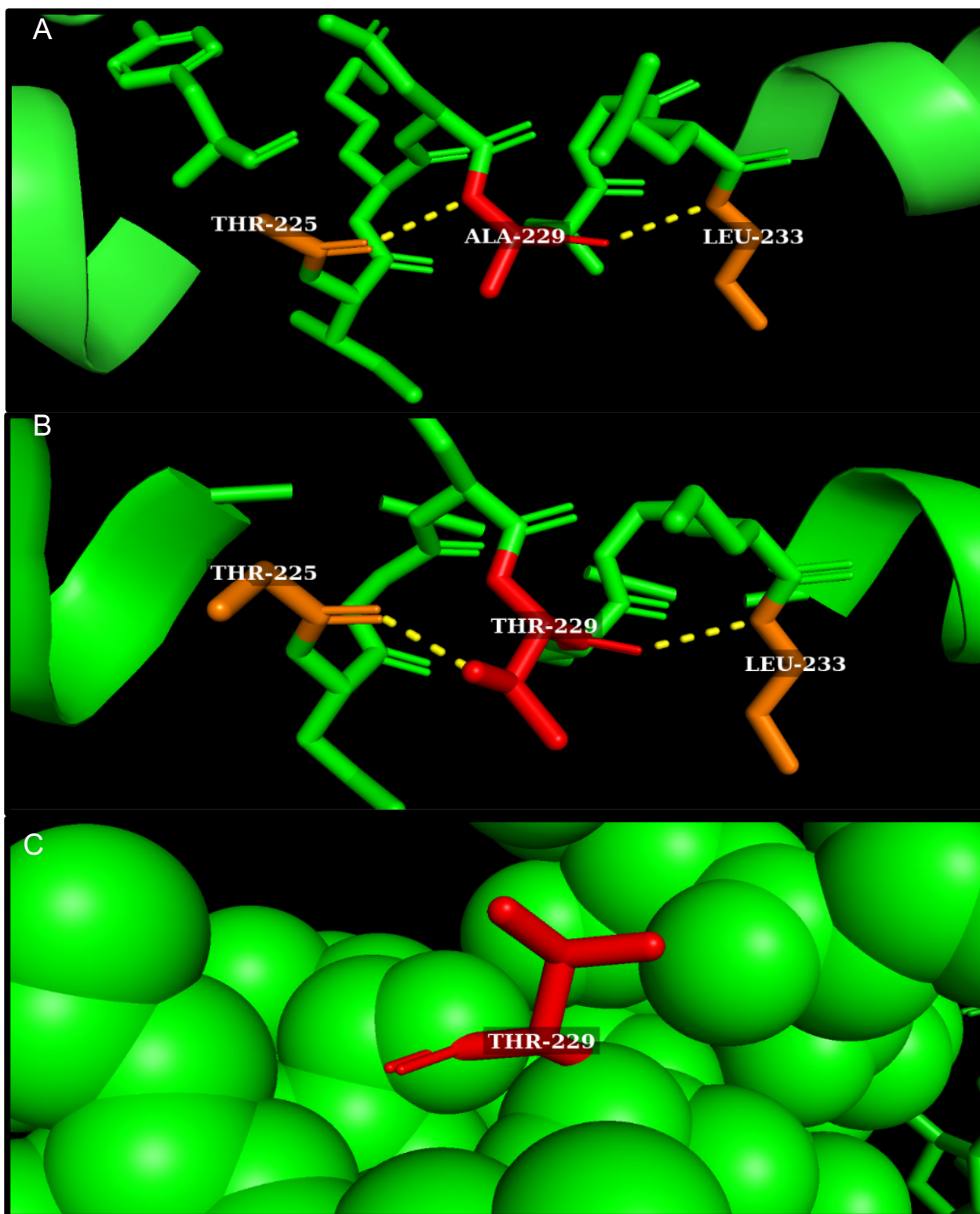

Supplement: S4 Fig — Both the wildtype Ala229 (red) (A) and the mutated Thr229 (red) (B) form two polar bonds (yellow dashed lines) with the surrounding amino acids (all in orange). However, the mutated Thr229 causes physical clashes with the surrounding amino acids (red) (C). (PDF) [file pone.0263606.s004.pdf]

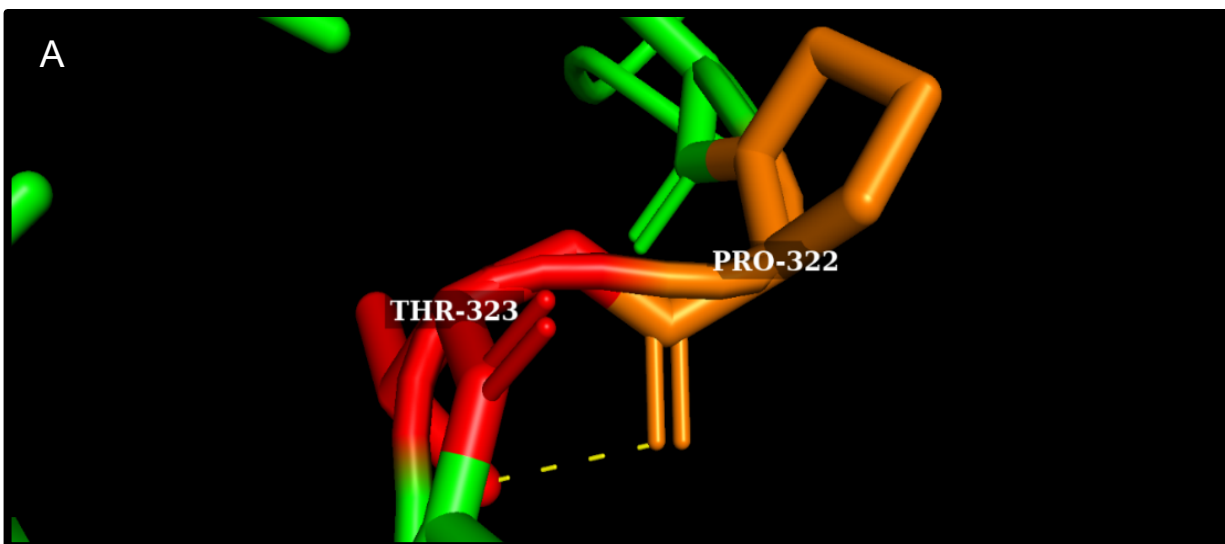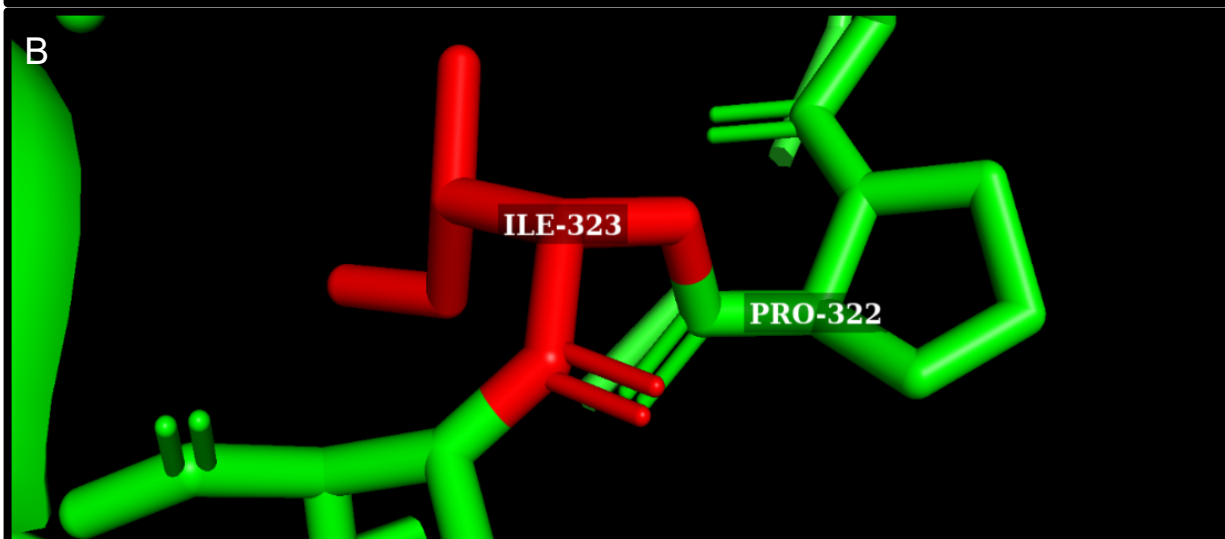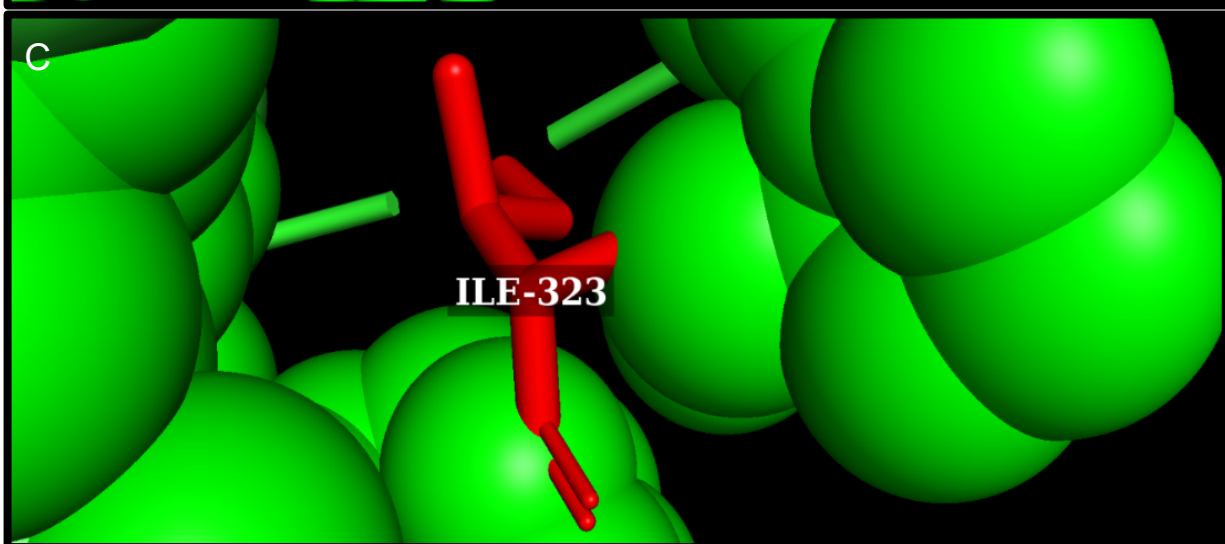

Supplement: S5 Fig — The wildtype Thr323 (red) (A) forms a single polar bond (yellow dashed line) with Pro322 (orange), while the mutated Ile323 (red) (B) shows a total loss of polar bonds and causes physical clashes with the surrounding amino acids (C). (PDF) [file pone.0263606.s005.pdf]
